# Obesity exacerbates influenza-induced respiratory disease via the arachidonic acid-p38 MAPK pathway

**DOI:** 10.3389/fphar.2023.1248873

**Published:** 2023-08-23

**Authors:** Ravishankar Chandrasekaran, Carolyn R. Morris, Isabella M. Butzirus, Zoe F. Mark, Amit Kumar, Dhemerson Souza De Lima, Nirav Daphtary, Minara Aliyeva, Matthew E. Poynter, Vikas Anathy, Anne E. Dixon

**Affiliations:** ^1^ Department of Medicine, Larner College of Medicine, University of Vermont, Burlington, VT, United States; ^2^ Pathology and Laboratory Medicine, Larner College of Medicine, University of Vermont, Burlington, VT, United States

**Keywords:** influenza A virus, obesity, p38 MAPK, arachidonic acid, lung inflammation, lung injury

## Abstract

Obesity is a risk factor for severe influenza, and asthma exacerbations caused by respiratory viral infections. We investigated mechanisms that increase the severity of airway disease related to influenza in obesity using cells derived from obese and lean individuals, and *in vitro* and *in vivo* models. Primary human nasal epithelial cells (pHNECs) derived from obese compared with lean individuals developed increased inflammation and injury in response to influenza A virus (IAV). Obese mice infected with influenza developed increased airway inflammation, lung injury and elastance, but had a decreased interferon response, compared with lean mice. Lung arachidonic acid (AA) levels increased in obese mice infected with IAV; arachidonic acid increased inflammatory cytokines and injury markers in response to IAV in human bronchial epithelial (HBE) cells. Obesity in mice, and AA in HBE cells, increased activation of p38 MAPK signaling following IAV infection; inhibiting this pathway attenuated inflammation, injury and tissue elastance responses, and improved survival. In summary, obesity increases disease severity in response to influenza infection through activation of the p38 MAPK pathway in response to altered arachidonic acid signaling.

## Introduction

Obesity is a risk factor for severe disease in response to many respiratory viruses (Flerlage et al., 2021) and obese people with asthma are more likely to have an asthma exacerbation when they develop a respiratory infection compared to lean people (Tang et al., 2019). Obesity was first identified as an independent risk factor for severe influenza during the 2009 H1N1 pandemic (Vaillant et al., 2009; Miller et al., 2010; Bassetti et al., 2011; Thompson et al., 2011; Van Kerkhove et al., 2011; Sun et al., 2016). Since then, the impact of obesity on disease severity and mortality has been recognized during seasonal influenza epidemics as well (Fezeu et al., 2011; Martin et al., 2013; Okubo et al., 2018; Cocoros et al., 2022). Increased airway disease related to influenza in obese people is a major public health issue as over 40% of the US population is obese; elevated disease severity related to viral infections is likely one of the major causes of the nearly five-fold risk of hospitalization for obese compared with lean people with asthma.

Some studies, using mice models of genetic and diet-induced obesity (DIO), have investigated the pathophysiology of increased influenza severity in obesity. While observations from these studies vary, likely due to differing models and viral strains, these studies suggests DIO causes delayed expression of cytokines and chemokines in response to IAV-PR8 (to model seasonal disease) (Smith et al., 2007), while pandemic H1N1 increases inflammation and mortality in obese mice (Milner et al., 2015; Easterbrook et al., 2011; Zhang et al., 2013; O'Brien et al., 2012). Obese mice also have a decreased Type I interferon response (Smith et al., 2007). Some studies have indicated that obesity may lead to increased lung damage caused by impaired wound healing leading to increased lung permeability and pulmonary edema (O'Brien et al., 2012; Karlsson et al., 2017). However, the mechanisms by which obesity mediates increased severity in influenza infection and its impact on airway hyperresponsiveness (AHR) is unclear.

Obesity is characterized by altered lipid metabolism and elevated circulating free fatty acids (Boden, 2008). Circulating long chain saturated fatty acids (SFA) and ω-6 polyunsaturated fatty acids (ω-6 PUFA) are elevated in obesity and contribute to inflammation (Raphael and Sordillo, 2013; Hammad and Jones, 2017; Wrzosek et al., 2022). Arachidonic acid (AA), a ω-6 PUFA, and its metabolites play a pro-inflammatory role in many diseases including asthma, arthritis, atherosclerosis and cancer (Rutting et al., 2018a; Wang et al., 2021). Recently, arachidonic acid and its metabolite prostaglandin E2 (PGE2) have been implicated in pro-inflammatory response during viral infection (Coulombe et al., 2014; Chandrasekharan et al., 2016; Chen et al., 2022). Arachidonic acid treatment increases cytokine production via MAPK signaling in response to the viral mimic poly (I:C) (Rutting et al., 2018b). Mitogen-activated protein kinases (MAPK) like c-Jun N-terminal kinase (JNK) and p38 MAPK, which are vital during immune response, also promote viral replication (Meineke et al., 2019). Specifically, p38 MAPK is needed for both production of inflammatory cytokines such as IL-1β and IL-6 and viral replication (Yu et al., 2020). P38 MAPK is also needed for synthesis of PGE2 from arachidonic acid and targeting p38 activation reduces IAV-induced PGE2 production (Mizumura et al., 2003; Li et al., 2020; Yang et al., 2022). However, the contribution of obesity to altered arachidonic acid metabolism and downstream MAPK signaling during influenza infection is not known.

The objective of our study was to investigate mechanisms contributing to severe airway disease in response to influenza in obesity. We used primary human nasal epithelial cells (pHNECs) isolated from lean and obese people, a DIO mice model and AA treatment of human bronchial epithelial cells (HBE) to study the impact of obesity on influenza infection. We found that CCL20 inflammatory cytokine production and PAI-1 injury mediator production increased in nasal cells from obese compared with lean individuals. Diet induced obesity in mice increased inflammation, injury and remodeling responses to IAV, which was associated with increased tissue elastance response to methacholine. IAV infection in obese mice and AA-treated HBE cells activated p38 MAPK; targeted inhibition of p38 MAPK decreased PGE2, and inflammation, injury and tissue elastance response. Collectively our results show that obesity contributes to increased inflammation and lung injury caused by influenza infection and that these effects are likely mediated by elevated arachidonic acid-p38 MAPK signaling.

## Results

### Primary nasal epithelial cells derived from obese individuals produce higher levels of inflammatory and injury markers following IAV infection

**FIGURE 1:**
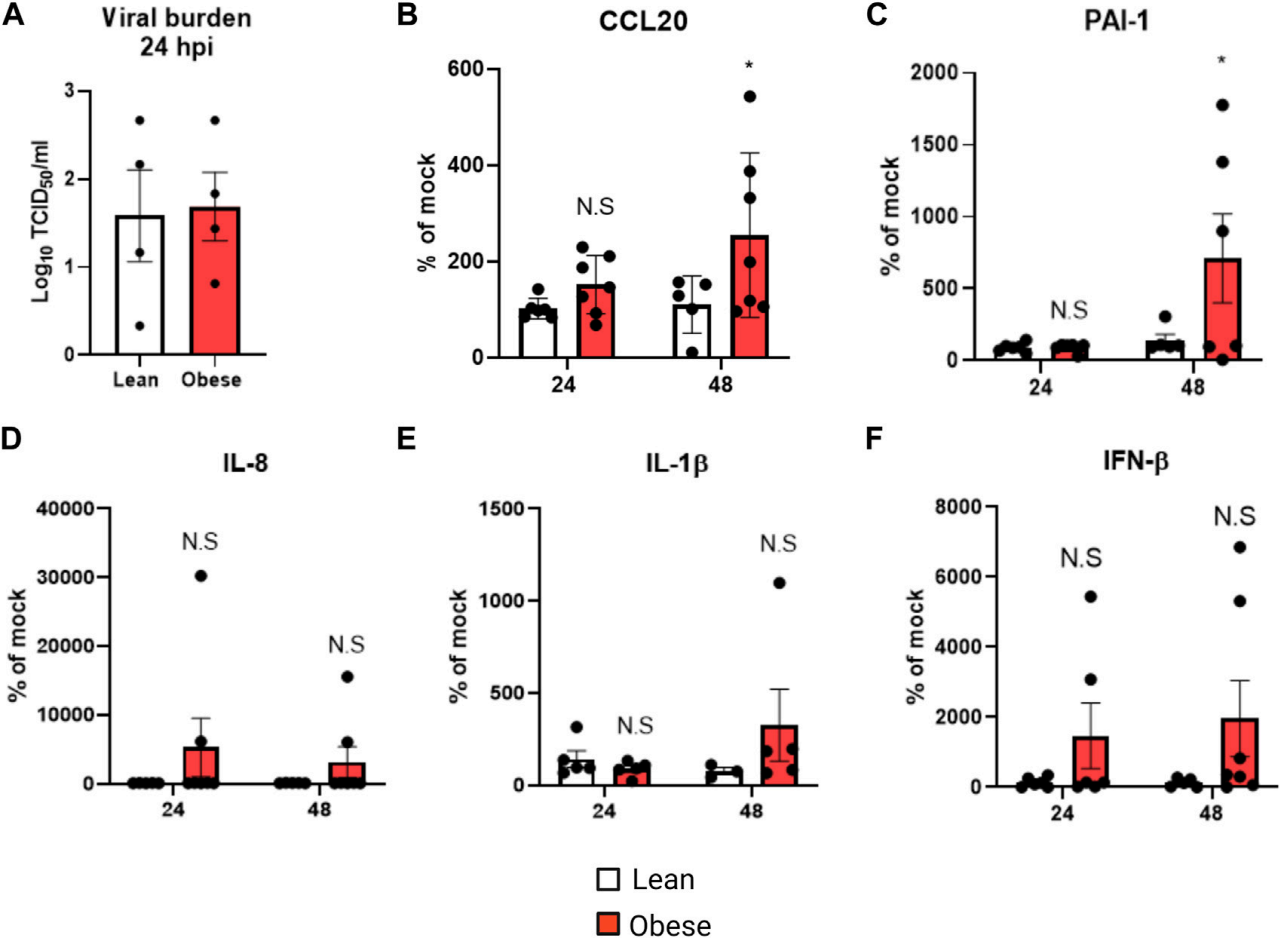
Elevated inflammatory and injury marker production in obese pHNECs infected with IAV: **(A)** TCID_50_ measurement of viral burden in lean and obese pHNECs (4 samples per group); **(B–E)**: **(B)** CCL20 **(C)** PAI-1 **(D)** IL-1β **(E)** IL-8 **(F)** IFN-β protein levels in the supernatant (4**–**7 samples per group). * Significant difference between lean and obese pHNECs, N.S, not significant; q values < 0.05 were regarded as discovery or statistically significant. Error bars ±SEM.

We used pHNECs derived from lean and obese individuals to investigate the effects of obesity on viral replication, and inflammatory and injury mediators. Viral burden, measured by TCID_50,_ was similar in cells obtained from lean and obese individuals (Figure 1A); however CCL20 (Figure 1B) and the injury mediator PAI-1 (Figure 1C) were significantly elevated in cells from obese individuals at 48 h post-infection (hpi). IL-8 (Figure 1D), IL-1β (Figure 1E) and IFN-β (Figure 1F) levels were elevated in cells from obese individuals in response to IAV, but this was not statistically significant. These results show that with similar levels of viral replication, cells from obese individuals produce higher levels of inflammatory and injury mediators compared to cells from lean individuals.

### IAV-PR8 exacerbates airway inflammation in obese mice

**FIGURE 2:**
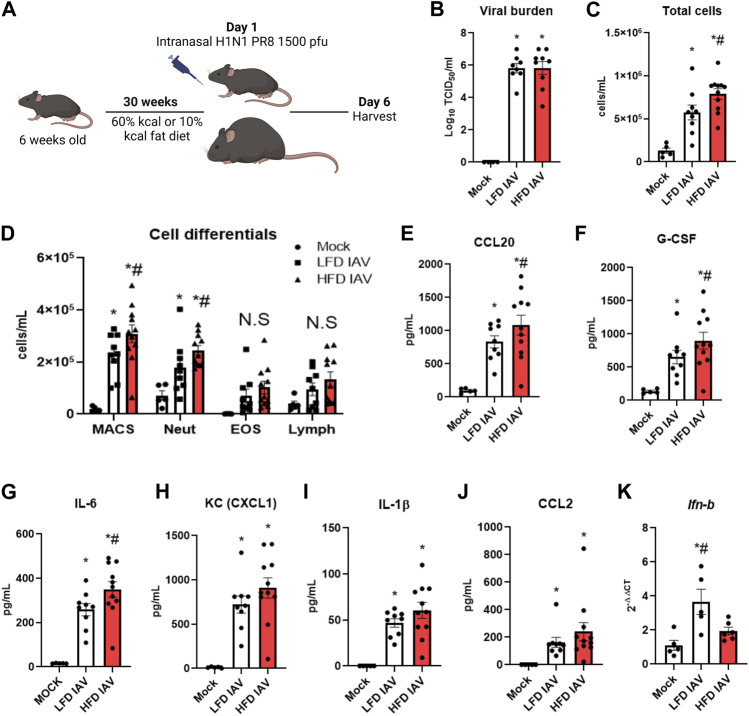
Elevated inflammatory parameters in HFD-IAV mice: **(A)** Schematic of DIO and IAV-PR8 exposure **(B)** TCID_50_ measurement of viral burden in the lungs; **(C,D)**: Cellular infiltration: **(C)** Total cells and **(D)** Cell differentials in the BAL; **(E–J)**: Cytokines in BAL **(E)** CCL20, **(F)** G-CSF, **(G)** IL-6 **(H)** KC **(I)** IL-1β and **(J)** CCL2 levels; **(K)** qRT-PCR for IFN-β. * Significant difference between Mock and IAV, # significant difference between LFD IAV and HFD IAV. q values < 0.05 were regarded as discovery or statistically significant. Error bars ±SEM.

To model obesity, we maintained male and female mice on a high fat diet (HFD) or low fat diet (LFD) for 30 weeks (10–11 months of age) before IAV-PR8 infection (Figure 2A). To assess viral burden, we measured TCID_50_ 6 days post infection (dpi), using lung tissue derived from the right inferior lobe. Viral burden was similar in lean and obese mice (Figure 2B). However, obese mice had increased bronchoalveolar lavage (BAL) total cell counts (Figure 2C), macrophages and neutrophils compared with lean mice (Figure 2D). BAL eosinophils and lymphocytes were similar in both diet groups (Figure 2D).

Innate cytokines were generally higher in obese than lean mice (Figures 2E–J): CCL20 (Figure 2E), G-CSF (Figure 2F) and IL-6 (Figure 2G) levels were significantly higher, while KC (Figure 2H), IL-1β (Figure 2I) and CCL2 (MCP1) (Figure 2J) levels were numerically higher, but not statistically significant, in obese mice. Type 1 interferon response, measured by qRT-PCR of *Ifn-β* (Figure 2K), was significantly lower in obese compared to lean mice. These results show that IAV-PR8 infection leads to exacerbated cellular inflammation and cytokine production, together with lower type 1 interferon response, in obese mice.

### IAV-PR8 exacerbates fibrosis, injury, stiffness, and airway elastance responsive in obese mice

**FIGURE 3:**
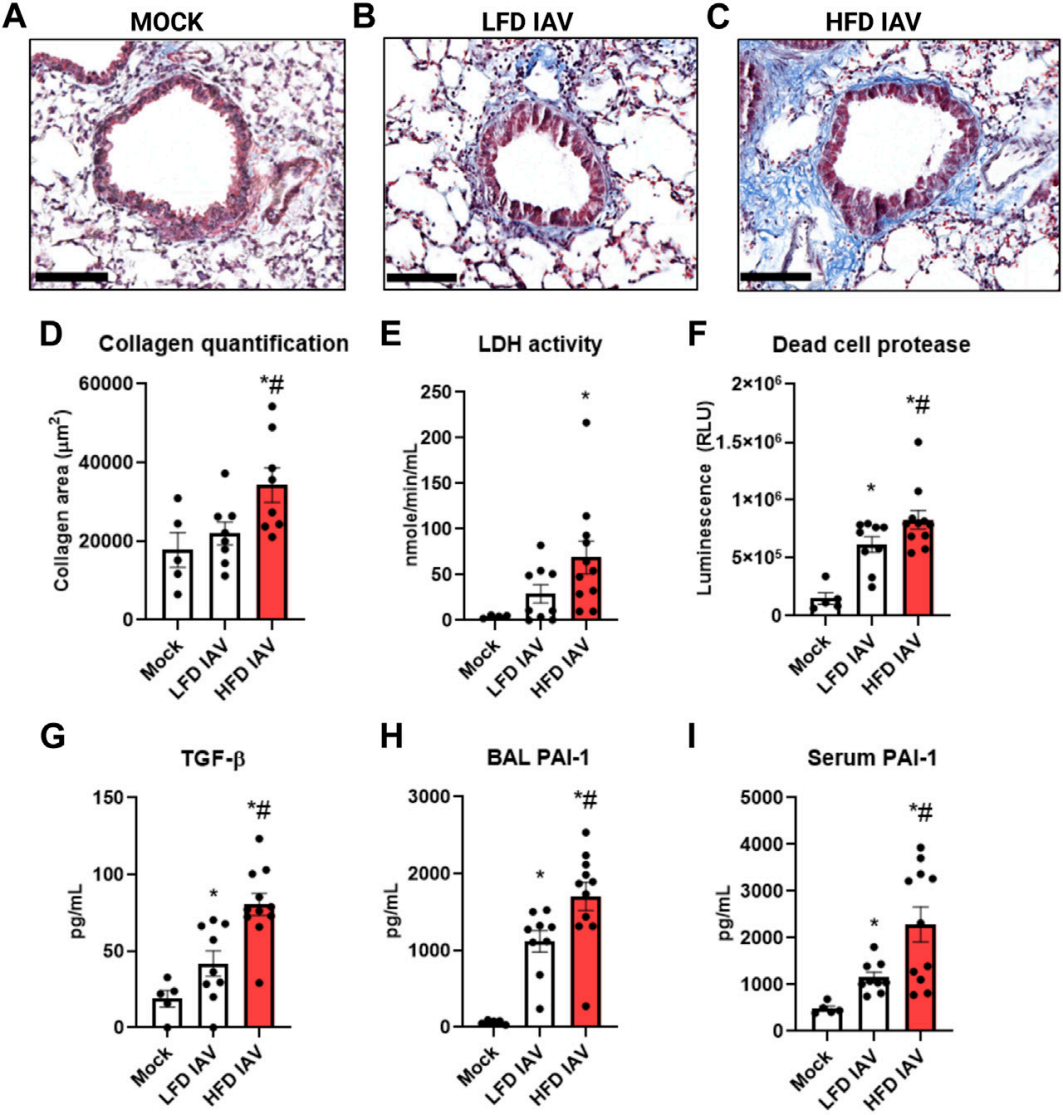
Elevated lung injury and peribronchial fibrosis in HFD-IAV mice: **(A–C)**: Representative images of Masson’s trichrome staining to measure collagen, Scale bars: 100 µM **(A)** MOCK **(B)** LFD IAV **(C)** HFD IAV; **(D)** Quantification of Masson’s trichrome staining; **(E–H)**: Lung injury parameters in BAL: **(E)** LDH activity **(F)** Dead cell protease activity **(G)** TGF-β protein level **(H)** PAI-1 protein level; **(I)** PAI-1 protein level in serum. * Significant difference between Mock and IAV, # significant difference between LFD IAV and HFD IAV. q values < 0.05 were regarded as discovery or statistically significant. Error bars ± SEM.

We assessed the fibrotic remodeling response to influenza infection in lean and obese mice. Peribronchial collagen deposition, measured by Masson’s trichrome staining, was higher in obese than lean infected mice (Figures 3A–D). Lung injury, assessed by BAL LDH (Figure 3E) and dead cell protease activity (Figure 3F), were also significantly elevated in obese mice. TGF-β (Figure 3G), an injury and fibrotic inducing growth factor, was significantly higher in obese mice. BAL (Figure 3H) and serum (Figure 3I) levels of the injury mediator PAI-1 were also elevated in obese mice.

**FIGURE 4:**
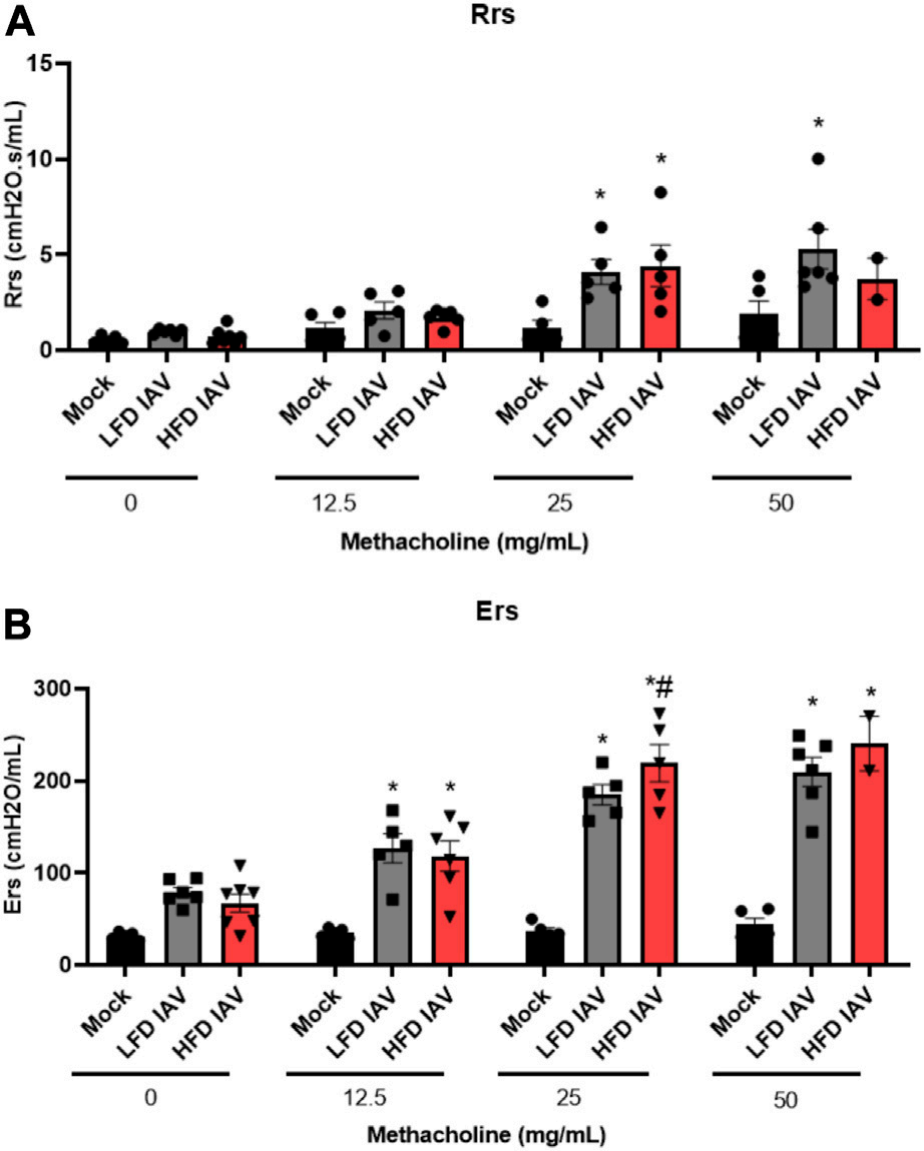
Airway reactivity in lean and obese IAV infected mice: **(A)** Resistance (Rrs) and **(B)** Elastance (Ers) (stiffness) as measured using flexivent. *Significant difference between Mock and IAV, # significant difference between LFD IAV and HFD IAV, q values < 0.05 were regarded as discovery or statistically significant. Error bars ±SEM.

Airway inflammation and injury induced by IAV infection can lead to airway reactivity and asthma exacerbations in humans. Therefore, we measured airway hyperresponsiveness (AHR) in response to methacholine. IAV-induced reactivity measured by respiratory system resistance (Rrs) (Figure 4A), which is primarily mediated by airway changes, increased in both lean and obese influenza infected mice at 25 mg/mL methacholine dose (Figure 4A). Tissue elastance (Ers) in response to IAV was increased in both lean and obese mice at 12.5 mg/mL. Ers levels at 25 mg/mL was significantly elevated in obese mice infected with IAV when compared to lean mice (Figure 4B). The highest dose of methacholine (50 mg/mL) resulted in spurious signals predominantly in the obese infected group, likely related to airway closure and mucus hypersecretion, which could not be best-fit to the single compartment linear regression model; hence a higher proportion of mice had to be excluded from HFD-IAV group compared to mock or the LFD-IAV group (Table 1). Our results show that IAV infection increases peribronchial fibrosis, airway injury, and tissue stiffness responses particularly in obesity.

**TABLE 1:** Proportion of mice that tolerated 50 mg/mL methacholine dose.

Group	Proportion
Mock	5/5 (100%)
LED IAV	6/7 (85.7%)
HFD IAV	2/7 (28.5%)

### Arachidonic acid increases IAV induced cytokine and injury markers, but decreases Type1 interferon response

**FIGURE 5:**
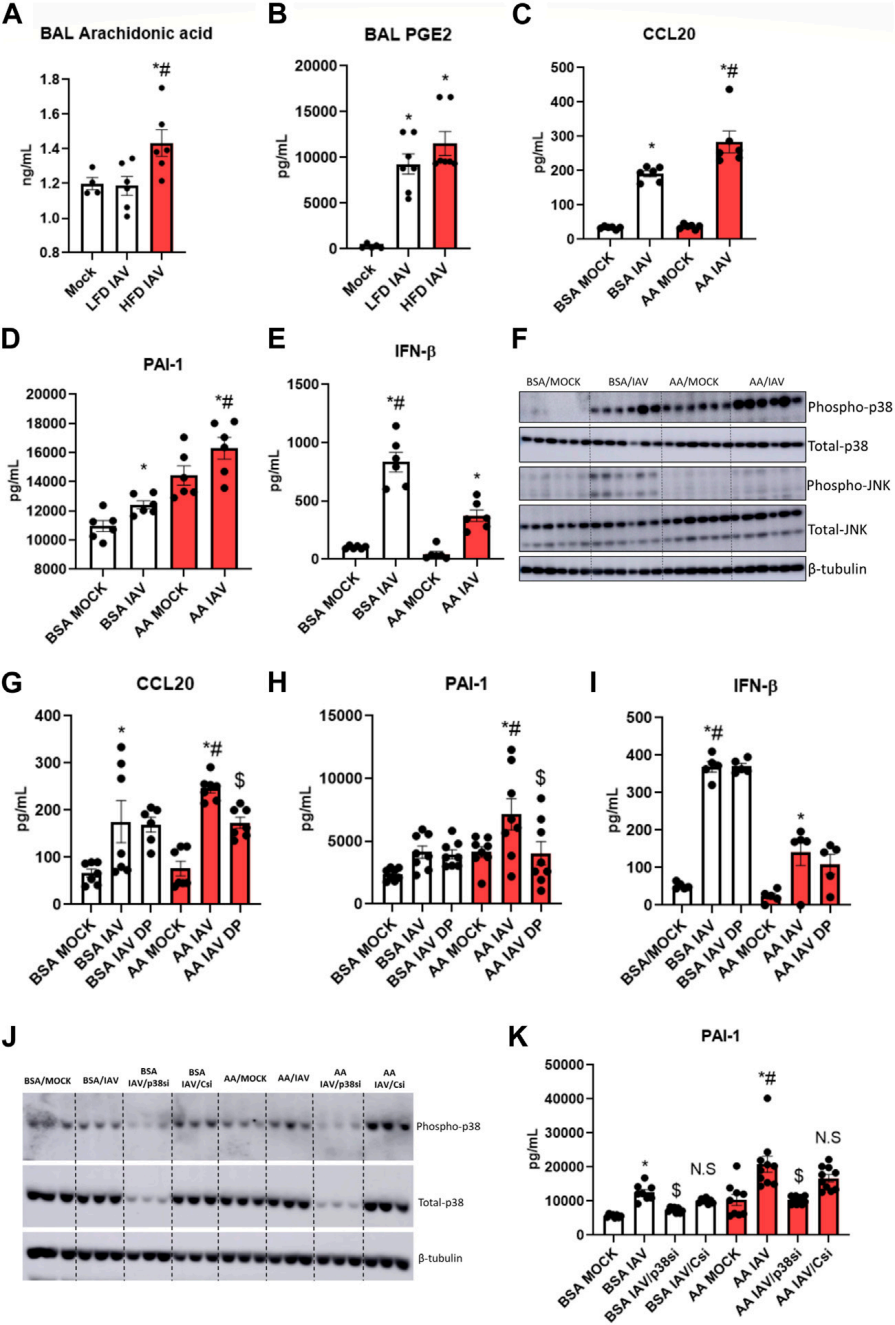
Arachidonic acid mediated inflammation, injury and p38 activation: **(A)** Arachidonic acid and **(B)** PGE2 levels in BAL, * significant difference between MOCK and IAV, # significant difference between LFD IAV and HFD IAV; **(C–E)**: Cytokine and injury marker levels in supernatant: **(C)** CCL20 **(D)** PAI-1 and **(E)** IFN-β; **(F)** Western blot to detect phospho-p38 and phospho-JNK1/2 levels; **(G–I)**: Effect of p38 MAPK inhibitor (DP) treatment on **(G)** CCL20 **(H)** PAI-1 and **(I)** IFNβ levels in supernatant. **(J)** Western blot to detect phospho and total-p38 MAPK following siRNA treatment; **(K)** Effect of p38 siRNA treatment on PAI-1 levels. * Significant difference between MOCK and IAV, # significant difference between BSA IAV and AA IAV, $ significant difference between AA IAV and AA IAV/DP or AA IAV/p38si. q values < 0.05 were regarded as discovery or statistically significant. Error bars ±SEM.

Elevated fatty acids and altered lipid metabolism are characteristic of obesity (Milner et al., 2015; Hammad and Jones, 2017). Metabolic profiling has shown increased lung arachidonic acid (AA) levels following influenza infection in obese mice (Milner et al., 2015). We measured arachidonic acid levels in the BAL of IAV-PR8 infected mice: AA was significantly elevated following IAV-PR8 infection only in HFD obese mice (Figure 5A). Next, we measured PGE2, an AA-metabolite implicated in IAV infection severity (Chen et al., 2022). In contrast, IAV-PR8 infection increased BAL PGE2 levels (Figure 5B) in lean mice, with a non-significant increase in HFD mice.

We investigated the role of arachidonic acid in IAV infection. We treated HBEC3-KT cells with AA or BSA-vehicle control for 4 h before infection with 2.5 multiplicity of infection (MOI) IAV-PR8. AA produced a significant increase in IAV-induced CCL20 (Figure 5C) and PAI-1 (Figure 5D), compared to BSA control. AA significantly decreased IFN-β (Figure 5E). These results suggest that elevated lung arachidonic acid following IAV infection might contribute to increased inflammation and injury, and reduced interferon response in obesity.

### Arachidonic acid increases p38 MAPK signaling, and inhibiting p38 MAPK reduces inflammatory markers

AA increases p38 and JNK MAPK signaling in lung fibroblasts in response to the viral mimetic poly (I:C) (Rutting et al., 2019). Hence, we investigated the effect of AA-IAV treatment on these pathways in HBEC3-KT cells. IAV-induced phospho-p38 levels were significantly elevated with AA compared to BSA control. While there was no detectable phospho-p38 in BSA-MOCK treated cells, there was a strong expression of phospho-p38 in AA-MOCK treated cells, indicating that p38 signaling is activated by arachidonic acid alone, and further increased by IAV infection (Figure 5F, Supplementary Figure S1A). In contrast to p38 MAPK, phospho-JNK1/2 levels were higher in BSA-IAV treated cells compared to AA-IAV treatment (Figure 5F, Supplementary Figure S1B).

To determine whether AA-IAV responses are mediated by p38 MAPK we pre-treated HBEC3-KT cells with dilmapimod (DP), a p38 specific inhibitor. DP-treatment significantly decreased AA-IAV induced CCL20 (Figure 5G) and PAI-1 (Figure 5H) while having no effects on IAV-induced IFN-β (Figure 5I). We also validated the effects of p38 MAPK attenuation by siRNA treatment. Pre-treatment of HBEC3-KT cells with *Mapk14* (p38 MAPK gene) but not control siRNA resulted in a decrease of both total and phospho-p38 MAPK levels (Figure 5J, Supplementary Figure S1C) leading to a concomitant decrease in AA/IAV-induced PAI-levels (Figure 5K). These results show that arachidonic acid increases p38 MAPK activation and inhibition of this pathway reduces AA-IAV induced inflammatory and injury responses *in vitro*.

### Targeting p38 MAPK reduces IAV-PR8 induced inflammation, injury, AHR and morbidity in obesity

**FIGURE 6:**
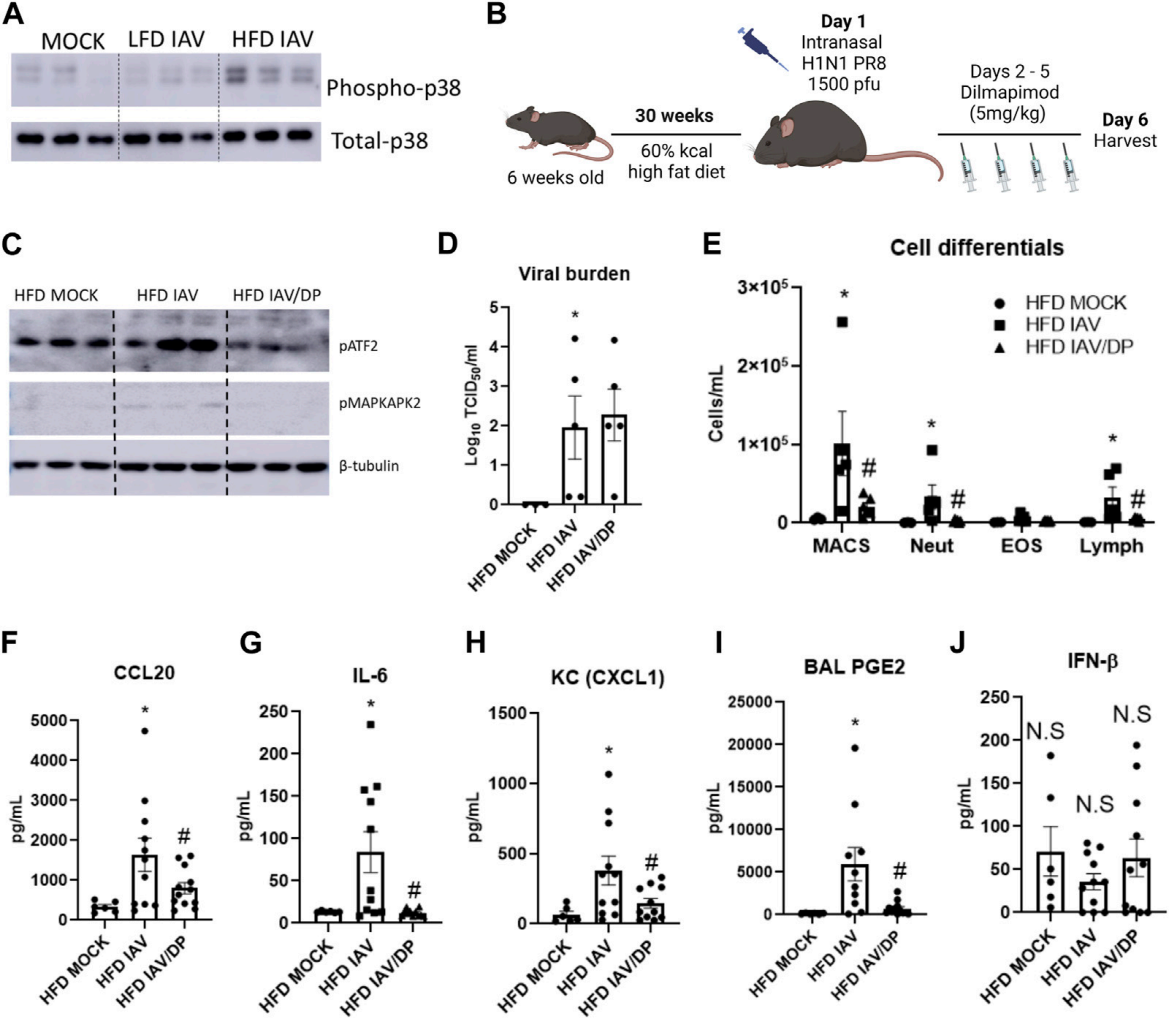
p38 MAPK inhibition reduces IAV-induced inflammation in HFD mice: **(A)** Western blot to detect phospho-p38 in the lungs; **(B)** Schematic of DIO, IAV-PR8 exposure and DP-treatment **(C)** Western blot to detect p38MAPK targets phospho-ATF2 and phospho MAPKAPK2; **(D)** TCID50 to measure viral burden; **(E)** Cell differentials in the BAL; F-I: Cytokines in the BAL: **(F)** CCL20 **(G)** IL-6 **(H)** KC; **(I)** PGE2 level in BAL; **(J)** IFN-β level in BAL. *Significant difference between HFD mock and HFD IAV, # significant difference between HFD IAV and HFD IAV/DP. q values < 0.05 were regarded as discovery or statistically significant. Error bars ±SEM.

Phospho-p38 MAPK levels were upregulated in obese-IAV compared to lean-IAV mice (Figure 6A, Supplementary Figure S1D). To investigate the role of p38 MAPK *in vivo*, we administered DP intraperitoneally for 4 consecutive days following IAV-PR8 infection and harvested mice 6 days post infection (Figure 6B). DP-treatment decreased the IAV-induced phosphorylation levels of p38 MAPK targets, ATF2 and MAPKAPA2 (Figure 6C, Supplementary Figures S1E, F). While DP-treatment did not have any effect on viral burden (Figure 6D), it significantly decreased IAV-PR8 induced macrophages, neutrophils and lymphocytes in obese mice (Figure 6E). DP-treatment also reduced IAV-PR8 induced CCL20 (Figure 6F), IL-6 (Figure 6G) and KC (Figure 6H). Previous studies have shown that MAPK play an important role in PGE2 release from bronchial epithelial cells (Mizumura et al., 2003). DP-treatment also reduced IAV-PR8 induced PGE2 levels in the BAL (Figure 6I). BAL IFN-β levels were not significantly elevated with IAV-treatment in obese mice and DP-treatment had no significant effect on IFN-β (Figure 6J). These results indicate that targeting p38 MAPK reduces IAV-induced inflammation in obese mice, but does not affect type-1 interferon responses.

**FIGURE 7:**
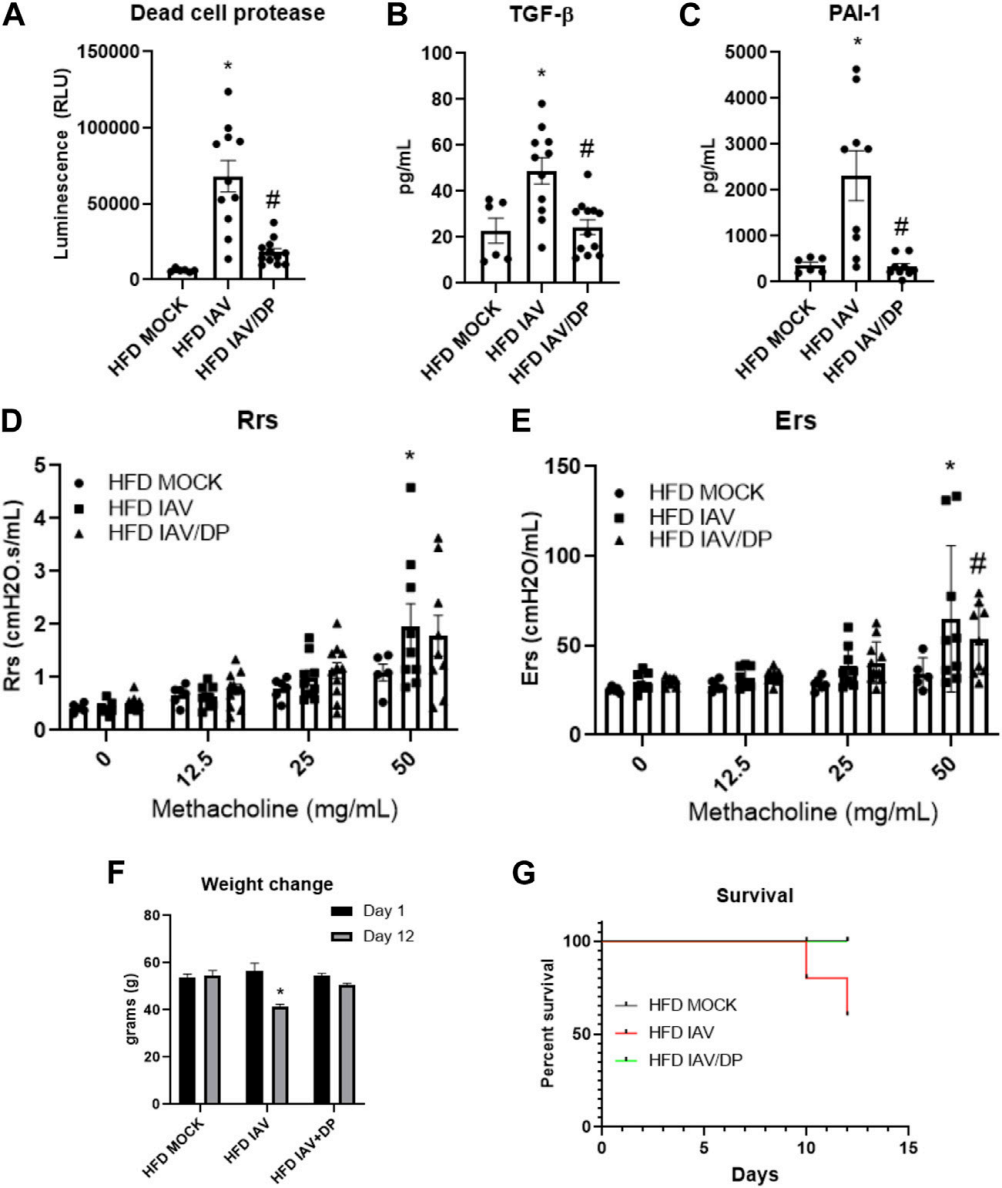
p38 MAPK inhibition reduces IAV-induced lung injury, lung stiffness, weight change and improves survival in HFD mice: **(A–C)**: Lung injury markers in BAL: **(A)** Dead cell protease activity **(B)** TGF-β **(C)** PAI-1; **(D)** Resistance (Rrs) and **(E)** Elastance (Ers) or stiffness as measured by flexivent; **(F)** Weight change (grams) 12 days post infection; **(G)** Percent survival 12 days post infection. *Significant difference between HFD mock and HFD IAV, # significant difference between HFD IAV and HFD IAV/DP. q values < 0.05 were regarded as discovery or statistically significant. Error bars ±SEM.

We then measured lung injury and airway hyperreactivity following DP-treatment in HFD-IAV mice. DP significantly reduced dead cell protease activity (Figure 7A), TGF-β (Figure 7B) and PAI-1 in response to IAV (Figure 7C). DP-treatment did not affect IAV-induced resistance responses (Rrs) (Figure 7D), however, it significantly reduced IAV tissue elastance response (Ers) (Figure 7E). To study the effects of inhibiting p38 MAPK on survival and morbidity, we administered DP after IAV-PR8 infection and followed the mice until 12 days post infection. Forty percent of obese mice infected with IAV-PR8 died, whereas no obese infected mice treated with DP died (Figure 7G). Obese DP-treated mice also had significantly less weight loss compared to surviving IAV-PR8 mice treated with vehicle (Figure 7F). Targeted inhibition of p38 MAPK might be a viable option to reduce IAV-induced inflammation, injury and airway reactivity in obesity.

## Discussion

Obesity is a risk factor for severe illness from respiratory viral infections, including influenza and COVID-19 (Flerlage et al., 2021). Obese people with asthma have increased morbidity related to respiratory infections. This increased risk related to viral infections in obesity has become a major public health issue as over 40% of the US population is obese and over 60% of people with severe asthma are obese. There are no therapies targeting influenza infection in obesity. The objective of our investigations was to investigate mechanisms that cause increased disease severity in response to influenza in obesity. We found that influenza infection in obesity is associated with increased airway inflammation, injury, peribronchial fibrosis and tissue-related airway reactivity that appear to be related, at least in part, to an increased arachidonic acid-p38 MAPK signaling pathway.

To accurately model obesity-associated pathophysiology of influenza infection we used 30-week mice fed a high fat diet. Like other reports, we found similar viral burden in the lungs of lean and obese mice (O'Brien et al., 2012). Interestingly, pHNECs infected with IAV also showed similar viral replication in both lean and obese individuals. However, even with similar levels of virus, obese mice show increased macrophage and neutrophil infiltration coupled with increased cytokine production. The inflammatory cytokine CCL20 and the lung injury mediator PAI-1 are produced by the airway epithelium in response to allergy, viral infections and other inflammatory conditions (Gómez-Melero and Caballero-Villarraso, 2023; Morrow and Mutch, 2023). It is noteworthy that both CCL20 and PAI-1 were also found to be elevated in pHNECs obtained from obese individuals, implicating an important role for these mediators in influenza infection in obesity.

The increased cytokine production was accompanied with a decrease in Type-1 interferon response in obese mice as reported elsewhere (Namkoong et al., 2019). We also observed increased lung injury and peribronchial fibrosis following influenza infection in obese mice. Exacerbated lung injury may lead to increased lung permeability which has been observed in obese mice during influenza infection (Karlsson et al., 2017). This was accompanied by abnormal lung function measured by elevated tissue elastance responses in obese mice. Inability to fit spurious signals as a consequence of exacerbated bronchoconstriction and mucus production to the single compartment model led to exclusion of many obese infected mice at the 50 mg/mL methacholine dose. Hence, the decline in lung function represented here underestimates the severity caused by obesity. These data suggest that the cumulative increase in inflammatory and injury mediators leads to a biologically significant effect on lung function following influenza infection in obesity.

In order to elucidate the immune signaling pathways contributing to increased IAV-induced inflammation and lung injury in HFD mice, we investigated NF-κB signaling, Leptin-STAT3, oxidized phospholipids and TRAIL-mediated apoptosis, pathways known to play important roles in obesity-related pathologies. However, while phospho-NF-κB, phospho-STAT3, oxidized phospholipids and TRAIL levels were upregulated with IAV-PR8 infection they were similar in both diet groups (data not shown).

Since obesity is characterized by nutritional imbalance, the impact of dietary fatty acids on the role of obesity in lung disease is important. We found that levels of arachidonic acid in the BAL were increased only in obese mice infected with influenza. AA-treatment augments poly (I:C) mediated cytokine production (Rutting et al., 2019). In this study we found that AA-treatment in human bronchial epithelial cells augments IAV-induced pro-inflammatory cytokines CCL20 and IL-8 as well as the lung injury marker PAI-1. Arachidonic acid is the primary fatty acid needed for the synthesis of eicosanoids which are important in inflammatory response (Wang et al., 2021). Influenza infection leads to increased prostaglandin E2 production (PGE2), a cyclooxygenase (COX-2) and prostaglandin E-synthase 1 (PGES1) catalyzed metabolite of arachidonic acid, which directly mediates cytokine production and impaired proliferation of alveolar macrophages thereby increasing viral replication (Coulombe et al., 2014; Chen et al., 2022). DIO in mice leads to an elevation of pandemic H1N1-induced PGE2 levels (Zhang et al., 2019a). In this study, we found that IAV-PR8 induced BAL PGE2 levels were slightly higher but not statistically significant in obese mice. Whether arachidonic acid metabolism leads to the specific upregulation of other eicosanoids following influenza infection in obesity needs to be explored.

IAV hijacks metabolic processes and intracellular signaling pathways to propagate and evade host immune responses (van de Sandt et al., 2012; Thaker et al., 2019). JNK kinases are needed for viral RNA synthesis, cytokine production and autophagy (Zhang et al., 2019b) while p38 MAPK is important in all stages of viral infection. P38 MAPK activation is needed for viral entry, viral RNA synthesis, ribonucleoprotein export and to prevent apoptosis of infected cells (Choi et al., 2016; Meineke et al., 2019). Previous studies have shown that p38 MAPK may also be involved in exacerbated cytokine response following influenza infection (Börgeling et al., 2014). Arachidonic acid treatment in human bronchial epithelial cells augments p38 MAPK activation following poly (I:C) treatment (Rutting et al., 2019). Here, we demonstrate that arachidonic acid treatment augments influenza induced p38 activation while it did not affect the activation of JNK 1 and 2. HFD in mice also augments p38 activation induced by IAV infection.

Substantial evidence both *in vitro* and *in vivo* show that targeting p38 MAPK activation with specific inhibitors provide anti-viral effects by reducing viral replication and attenuating host cytokine response (Börgeling et al., 2014; Choi et al., 2016; Growcott et al., 2018). Non-specific inhibitors that reduce p38 MAPK activation also reduce IAV induced inflammation and lung injury (Li et al., 2020; Zhou et al., 2020; Yang et al., 2022). Dilmapimod (DP) has been investigated in clinical trials of COPD and ARDS (Singh et al., 2010; Yang and Dumitrescu, 2017). In the current study, we show that targeted attenuation of p38 MAPK using DP attenuates pro-inflammatory cytokine and lung injury marker production in both the AA-IAV treatment in HBE cells as well as IAV infection in obese mice. Furthermore, DP-treatment attenuated IAV-induced cellular infiltration and airway reactivity in obese mice. Consequentially, p38 inhibition reduced weight loss and improved survival. The downstream mediator of p38 signaling during IAV infection is not clear; P38 MAPK signaling is important for PGE2 production (Mizumura et al., 2003; Li et al., 2020), and genetic deletion of *Cox2* as well as PGE2 receptor inhibition have been shown to reduce the severity of influenza infection (Carey et al., 2005; Chen et al., 2022). Here, we have shown that inhibition of p38 MAPK signaling reduces PGE2. Further experiments are needed to elucidate whether PGE2 is the primary effector mediating inflammatory and injury responses downstream of p38 MAPK during influenza infection.

There are limitations to the present work. The arachidonic acid (AA) treatment model in bronchial epithelial cells while not completely recapitulating the effects of obesity *in vivo*, upon IAV infection increases p38 MAPK activation leading to enhanced inflammation and injury markers with a concomitant decrease in Type 1 interferon response. Another limitation of the present work is the short time period to study influenza infection dynamics. This is especially important with respect to lung injury and fibrosis, which have a longer time course of emergence and resolution. Future time course experiments in the DIO mice model will potentially reveal novel pathological features of influenza infection in obesity. It would also provide insights into the temporal pattern of p38 activation and the effect of its inhibition on influenza induced inflammation, injury and fibrosis in obesity. Finally, the role of obesity during influenza infection is multifactorial and mediated by the cumulative effects of small but significant increases in inflammatory and injury mediators. While we have identified a significant role for some inflammation and injury mediators in this study, extensive measurements of cytokine/chemokines using luminex-based methods and omics-based identification of host-response pathways would provide better understanding of the pathophysiological role of obesity during influenza infection. In summary, we have shown that obesity exacerbates inflammation, injury, peribronchial fibrosis and lung stiffness responses caused by influenza infection. The effects of obesity are mediated by the activation of p38 MAPK signaling which may be caused by elevated arachidonic acid levels in the lung. Hence, targeting arachidonic acid metabolism and p38 MAPK signaling might be a viable option for treating severe influenza in obesity.

## Materials and methods

### Cell culture

HBEC3-KT human bronchial epithelial cells were cultured in Dulbecco’s modified Eagle’s medium (DMEM/F12) (Thermofisher scientific, Cat.No: 11330057) supplemented with growth factors. Arachidonic acid (AA) treatment was performed as described before (Rutting et al., 2019). Briefly, 80%–90% confluent HBE cells were growth factor starved for 2 h before treatment with 200 µM AA conjugated to BSA for 4 hrs. The media was removed and the cells were washed with PSB and replaced with fresh starvation media. The cells were then infected with 2.5 multiplicity of infection (MOI) IAV-PR8 for 4h after which the media was removed, the cells washed with PBS and replaced with fresh starvation media. After 20 h, the supernatants were collected to measure cytokine and injury marker levels. The cells were harvested with a buffer containing 20 mM Tris HCl (ph 7.5), 150 mM NaCl, 0.5% Igepal Ca-630, 10% glycerol, protease inhibitor cocktail (Sigma-Aldrich, P8340) and phosphatase inhibitor cocktails 1 and 2 (Sigma-Aldrich, P5726, P0044). For DP treatments, 1 μM DP was added and incubated for 1 h before the addition of AA.

### Human studies

The protocol was reviewed by the University of Vermont Institutional Review Board, and informed consent was obtained from all participants. Participants with obesity had a body mass index of 30 kg/m^2^ or more, lean participants had a body mass index between 18.5 and 24.9 kg/m^2^. Participants has no recent symptoms of respiratory tract infection, no chronic sinonasal symptoms, and were non-smokers.

Nasal cells were obtained from the inferior turbinats using a nasal cytology brush, then cultured in media containing equal proportions of DMEM and bronchial epithelial cell growth basal medium [BEBM; Lonza (cat no: CC-3171)] supplemented with bronchial epithelial singlequots kit [BEGM; Lonza (cat no: CC-4175)] and growth factors. The cells were expanded in culture for 1 passage and frozen. For experiments, the cells were revived in T-25 tissue culture flasks using the media mentioned above, and further cultured in 12 well plates. The cells were infected and harvested 24 and 48 h post infection as described for HBEC-3KT cells. 4-7 individual human samples with at least 3 biological replicated per sample were used for all assay.

### Mice

All mice experiments were approved by the University of Vermont Institutional animal care and use committee (IACUC protocol No: PROTO202000069). These experiments were performed as part of a larger study, which is beyond the scope of the current paper. Female C57BL/6 mice were obtained from Charles River laboratories and male C57BL/6 obtained from Jackson laboratories were used for all experiments. 4 week old mice were maintained on 60% kcal high-fat (Research diets D12492) or 10% low-fat (Research diets D12450B) diet for 30 weeks before experiments. A separate cohort of male mice were used for AHR measurements. 1500 plaque forming units (pfu) IAV H1N1 PR8 virus was used to infect mice intranasally on day1 and the BAL and lung tissues were harvested on day 6. For p38 inhibition, 5 mg/kg DP dissolved in DMSO and corn oil was administered intraperitoneally on days 2–5.

### Flow cytometry for cell counts

The identification of BAL immune cells were identified using hematoxylin and eosin staining on cytospin preparations or flow cytometry with specific cell surface markers as described elsewhere (Chandrasekaran et al., 2023).

### Masson’s trichrome staining

Collagen deposition was measured by Masson’s trichrome staining. Paraffin-embedded tissue sections were deparaffinized and fixed in Bouin’s mordant solution for 1 h at 56°C. After staining with Weigert’s hematoxylin, the slides were washed in running water followed by staining with Biebrich scarlet acid fuchsin solution for 2 min. After washing, the slides were then stained in phosphomolybdic/phosphotugstic acid and finally stained in aniline blue and 1% acetic acid, dehydrated and mounted using permaslip.

### ELISA

BAL was used to measure cytokine and chemokine levels. KC (CXCL1), G-CSF, CCL20, IL6, IL-1β, IFN-β, PAI-1 and TGF-β (R&D systems) was performed on 50 μL BAL following manufacturers’ protocol. Briefly, high binding ELISA plates were coated with capture antibody overnight at room temperature. After blocking with 1% BSA in PBS (0.5% Tween-20 in PBS for TGF-β), samples and standards were incubated overnight at 4°C. After washing, the plates were incubated with secondary antibody for 1 h, washed and incubated with streptavidin-HRP for 1 h at room temperature. TMB was used for detection and absorbance was measured at 450 nm. Background absorbance was measured at 540 nm.

### Western blot

25 µg protein lysates were loaded on a 12% SDS-PAGE. The proteins were transferred to a PVDF-membrane, blocked with 5% BSA and probed with the following antibodies: phospho-p38 MAPK (Cell signaling; Cat No: 9211), total-p38 MAPK (Cell signaling; Cat No: 9212), phospho-JNK (Cell signaling; Cat No: 9251), total JNK (Cell signaling; Cat No: 9252), β-tubulin (Abcam, AB6046). For cell culture experiments, p38 MAPK and JNK phosphorylation was detected 24 h post-infection with IAV-PR8.

### Lactate dehydrogenase (LDH) and dead cell protease assay

LDH substrate was combined with BAL and the resulting reduction of NAD to NADH was detected at 450 nm using the manufacturer’s protocol (Sigma-Aldrich (Cat No: MAK066). To assess dead cell protease activity, BAL was incubated with AAF-aminoluciferin substrate, and the luminescence generated by the breakdown product aminoluciferin was measured (Promega, Cat No: G9290).

### siRNA treatment

*Mapk14* SMARTPool siRNA (Cat.No: 003512-00-0005) and ON-TARGETplus non-targeting control siRNA (Cat.No: 001810-10-05) were obtained from Horizon discovery Ltd. HBEC3-KT cells were transfected with 25 nM siRNA using DharmaFECT transfection reagent (Horizon discovery Ltd., Cat.No T-2001-01) following the manufacturers’ protocol. The cells were incubated for 72 h to allow for maximal knockdown efficiency. Following this, the cells were treated with AA/BSA and infected with IAV-PR8 as described above.

### AHR measurements

The mice were anesthetized through the intraperitoneal injection of sodium pentobarbital at a dosage of 90 mg/kg, followed by the administration of pancuronium bromide, which is a paralyzing agent. The mice were then ventilated using flexivent (SCIREQ) and AHR was induced using aerosolized methacholine. The values of Newtonian resistance (Rn), tissue damping (G), and tissue elastance (H) were taken every 10 s for a period of 3 min, resulting in a total of 18 measurements. To calculate the average, any values with a coefficient of determination (COD) less than 0.85 were excluded.

### Statistical analysis

Experiments were conducted in both male and female mice and the resulting data was combined and analyzed using appropriate statistical methods such as one or two-way analysis of variance (ANOVA) and a two-stage linear step-up proceduce of Benjamini, Krieger, and Yekutieli test to account for multiple comparisons. The mean values ±standard error of mean (SEM) were used to express the data for all results, and q-values <0.05 were considered statistically significant. The ROUT method was employed with a false discovery rate cutoff of Q = 1% to detect and eliminate outliers from analysis. Graphpad Prism 8 (GraphPad software Inc., CA) was used to create statistical analysis and plots. Detailed description of the statistical analysis is included in Supplementary Table S1. All figures created/assembled with biorender.com.

## Data Availability

The original contributions presented in the study are included in the article/Supplementary Material, further inquiries can be directed to the corresponding authors.
