# Supplementary material for: Obesity exacerbates influenza-induced respiratory disease via the arachidonic acid-p38 MAPK pathway
Source: Front Pharmacol. 2023 Aug 23;14:1248873. doi: 10.3389/fphar.2023.1248873 (PMC10482034; doi:10.3389/fphar.2023.1248873)
Supplement: Supplementary file 1 [file Presentation1.PPTX]

## Slide 1
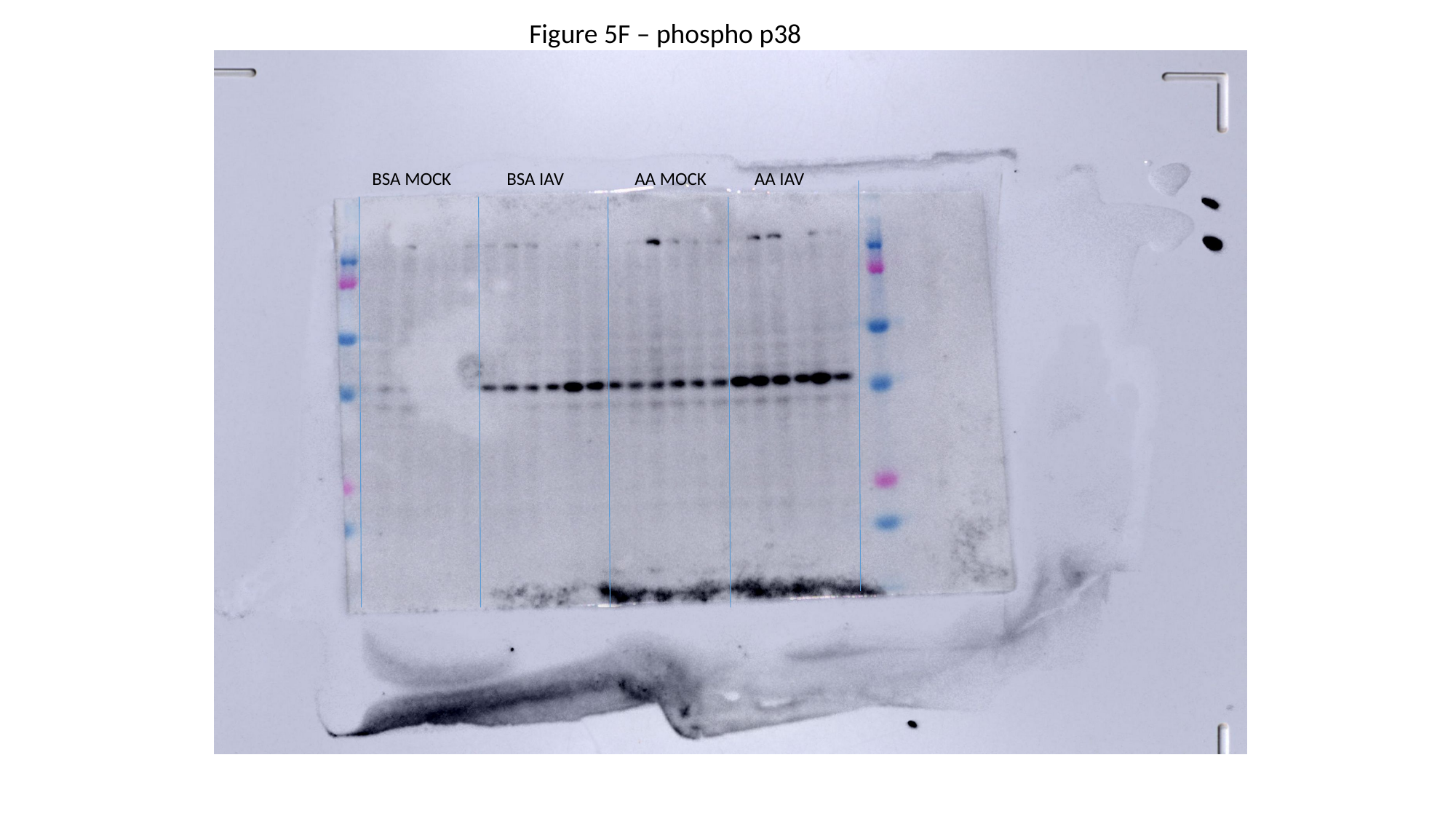

Figure 5F – phospho p38
AA IAV
AA MOCK
BSA IAV
BSA MOCK

## Slide 2
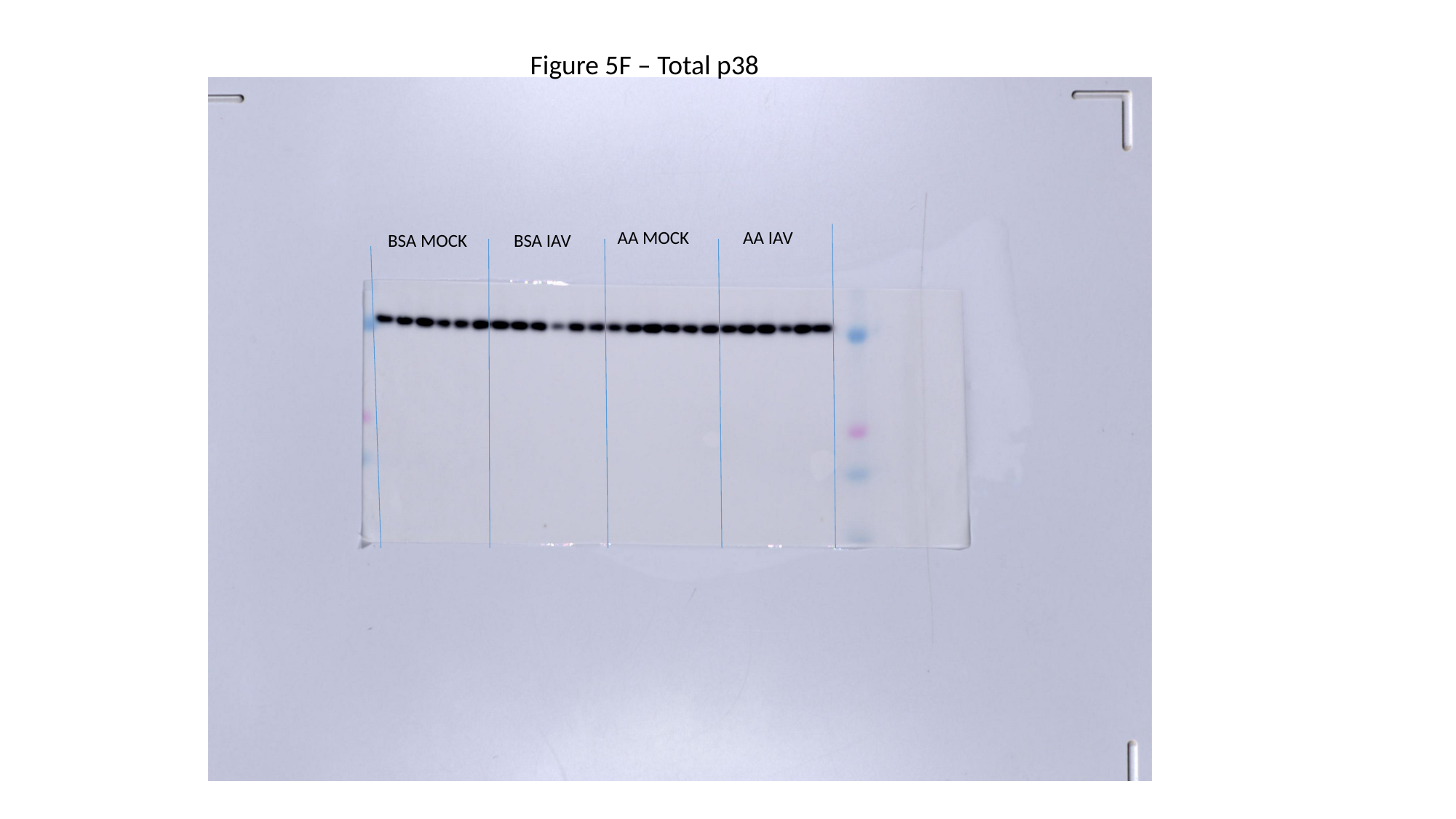

Figure 5F – Total p38
AA MOCK
AA IAV
BSA MOCK
BSA IAV

## Slide 3
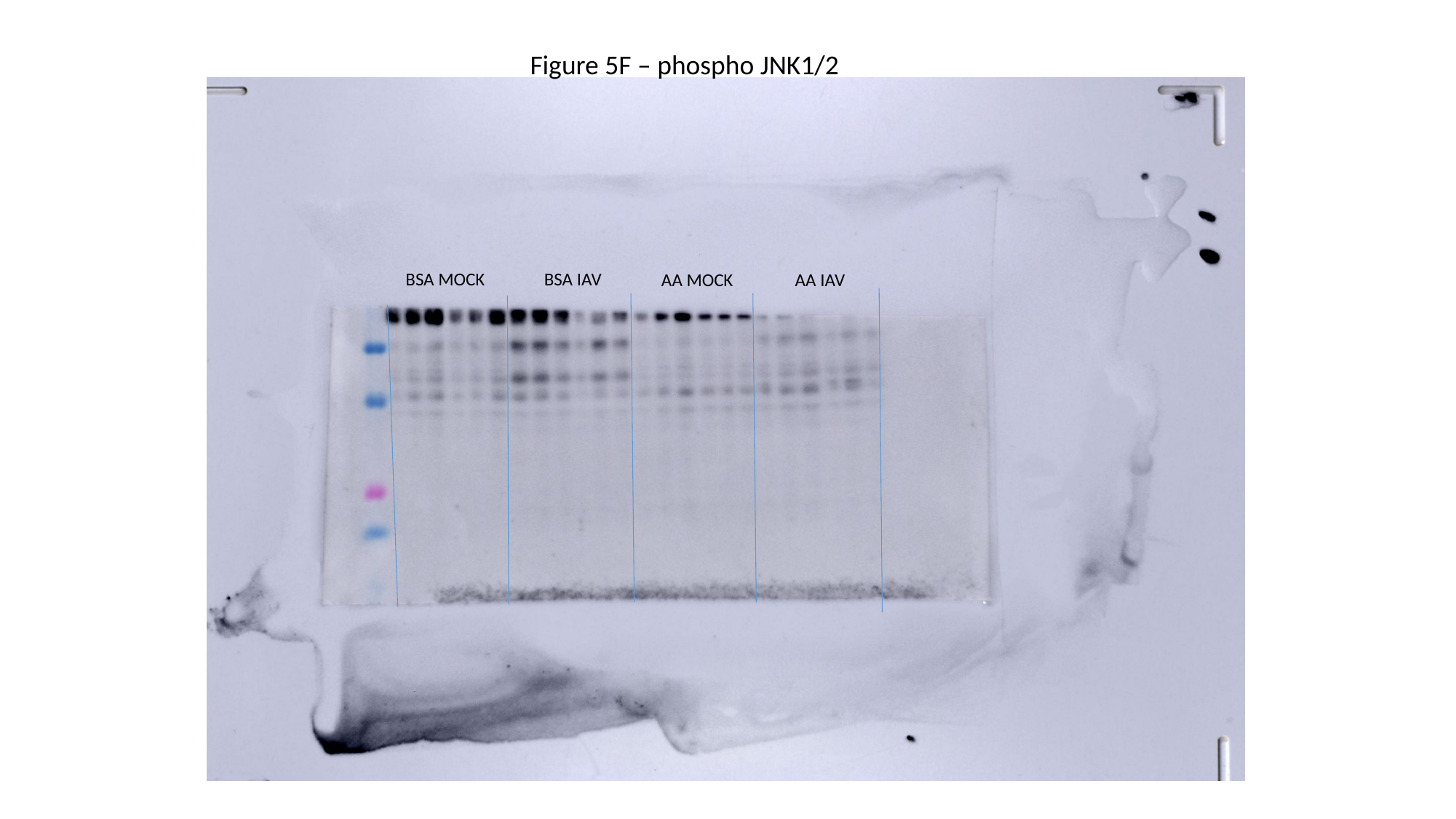

Figure 5F – phospho JNK1/2
BSA MOCK
BSA IAV
AA MOCK
AA IAV

## Slide 4
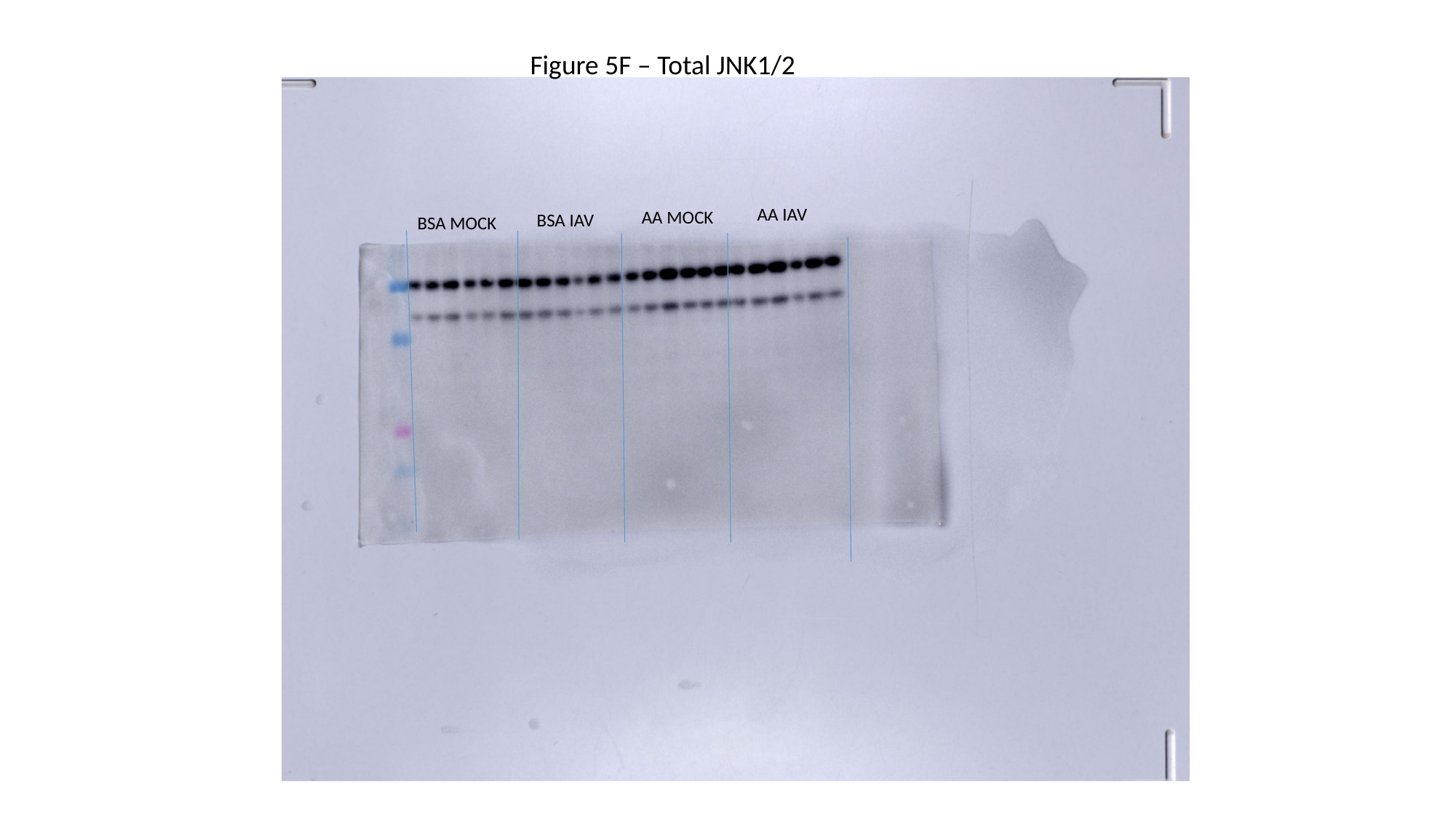

Figure 5F – Total JNK1/2
AA IAV
AA MOCK
BSA IAV
BSA MOCK

## Slide 5
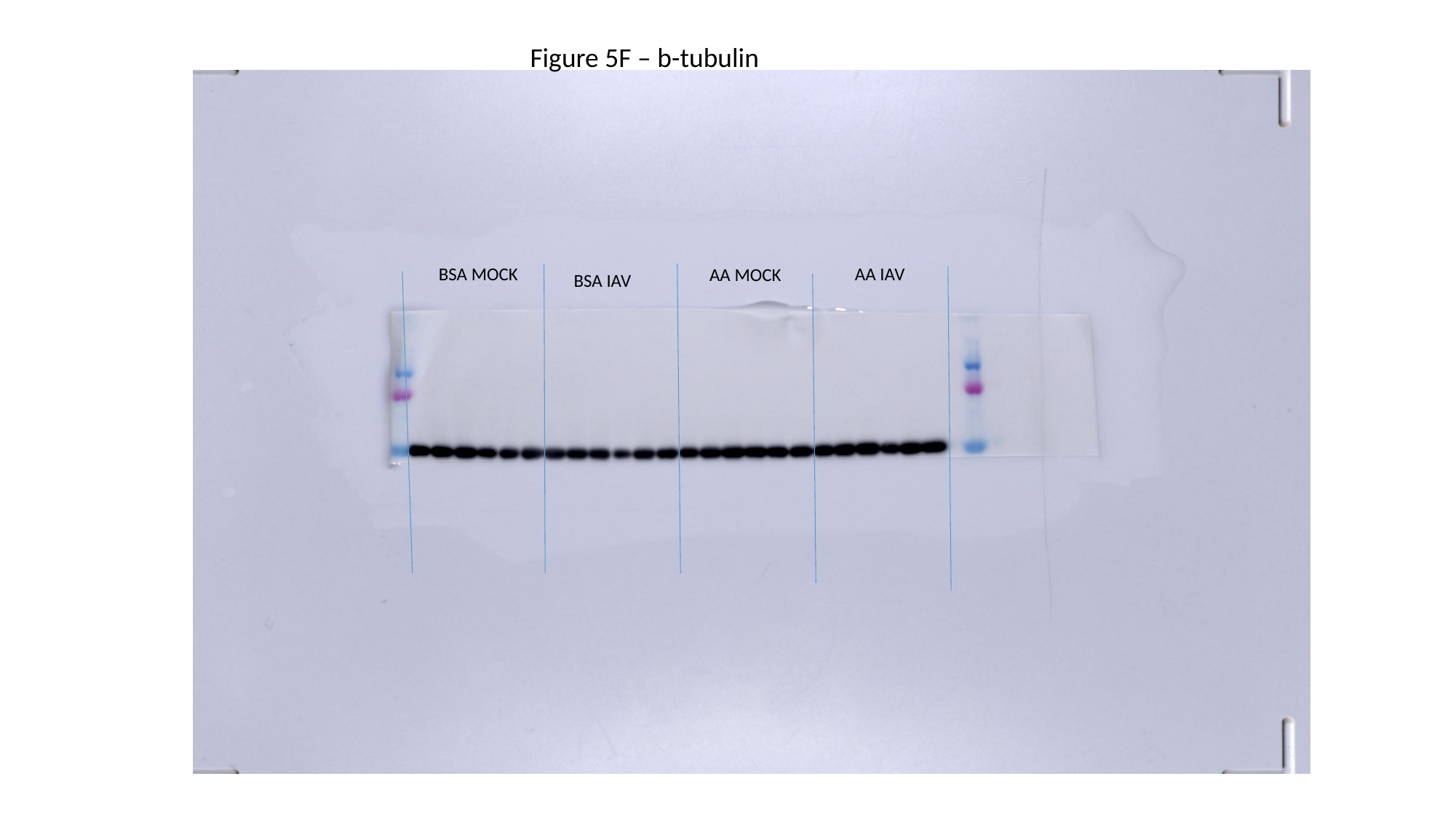

Figure 5F – b-tubulin
AA IAV
BSA MOCK
AA MOCK
BSA IAV

## Slide 6
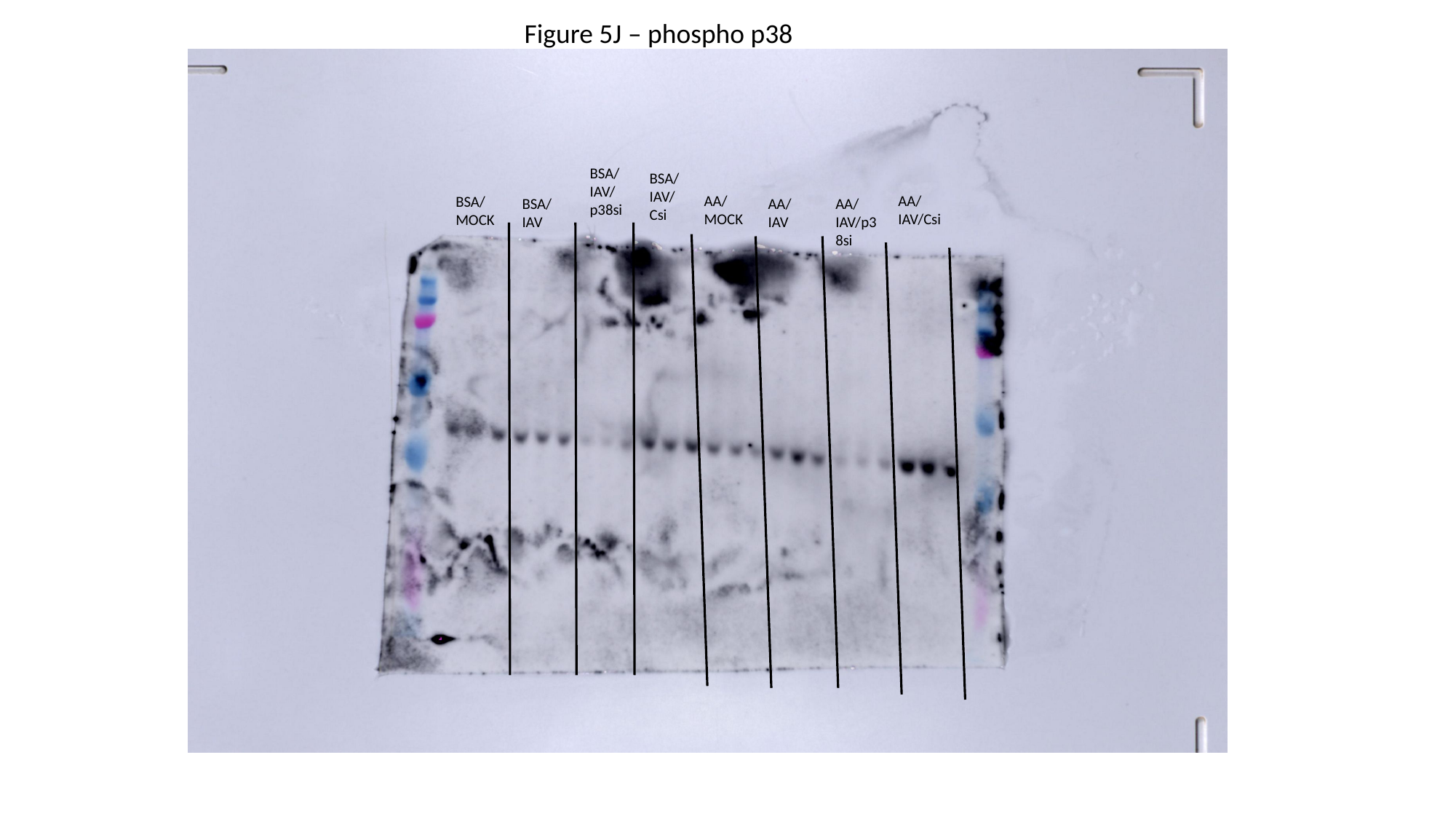

Figure 5J – phospho p38
BSA/IAV/p38si
BSA/IAV/Csi
AA/ MOCK
AA/ IAV/Csi
BSA/ MOCK
BSA/IAV
AA/ IAV
AA/ IAV/p38si

## Slide 7
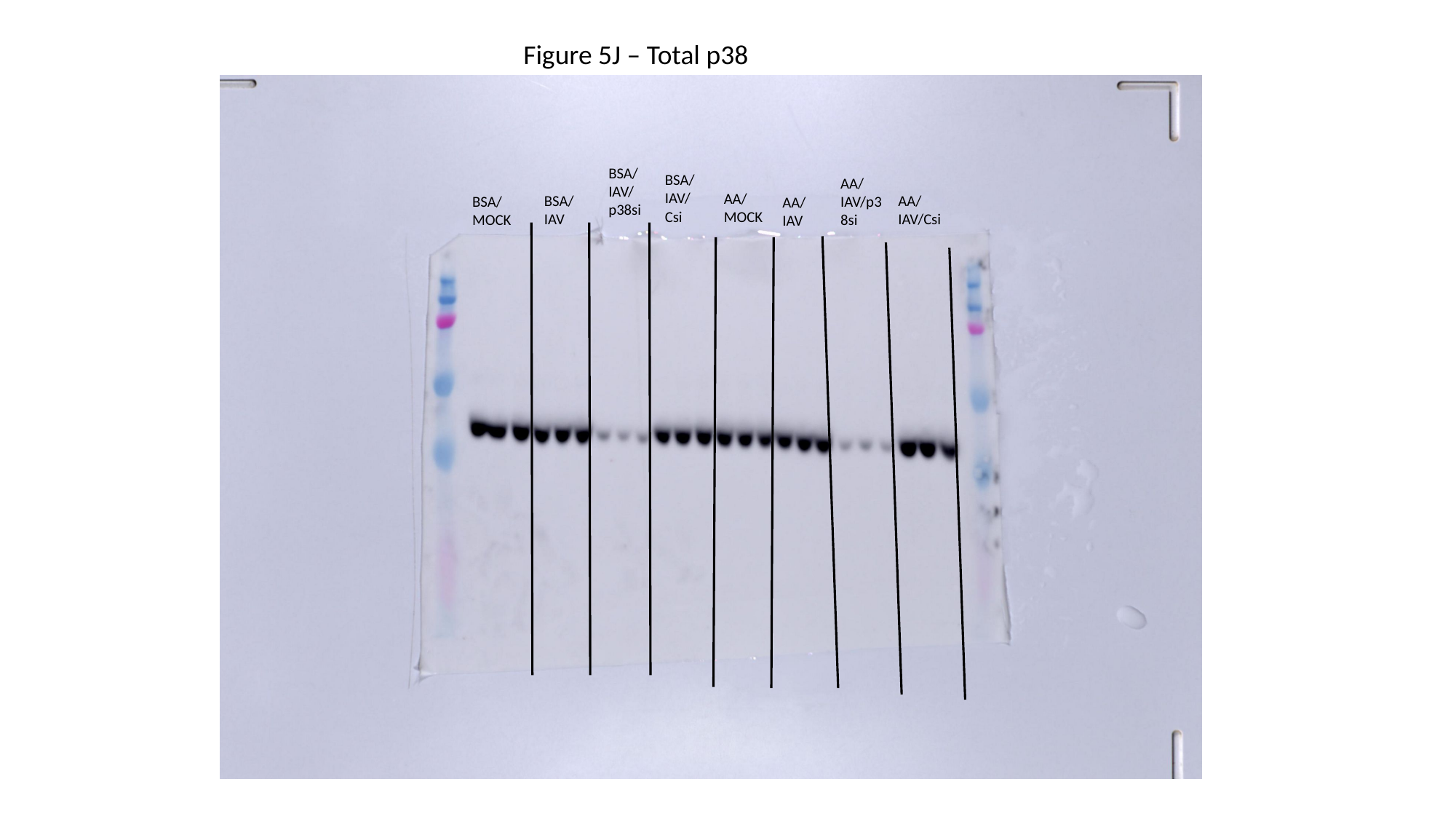

Figure 5J – Total p38
BSA/IAV/p38si
BSA/IAV/Csi
AA/ IAV/p38si
AA/ MOCK
BSA/IAV
AA/ IAV/Csi
BSA/ MOCK
AA/ IAV

## Slide 8
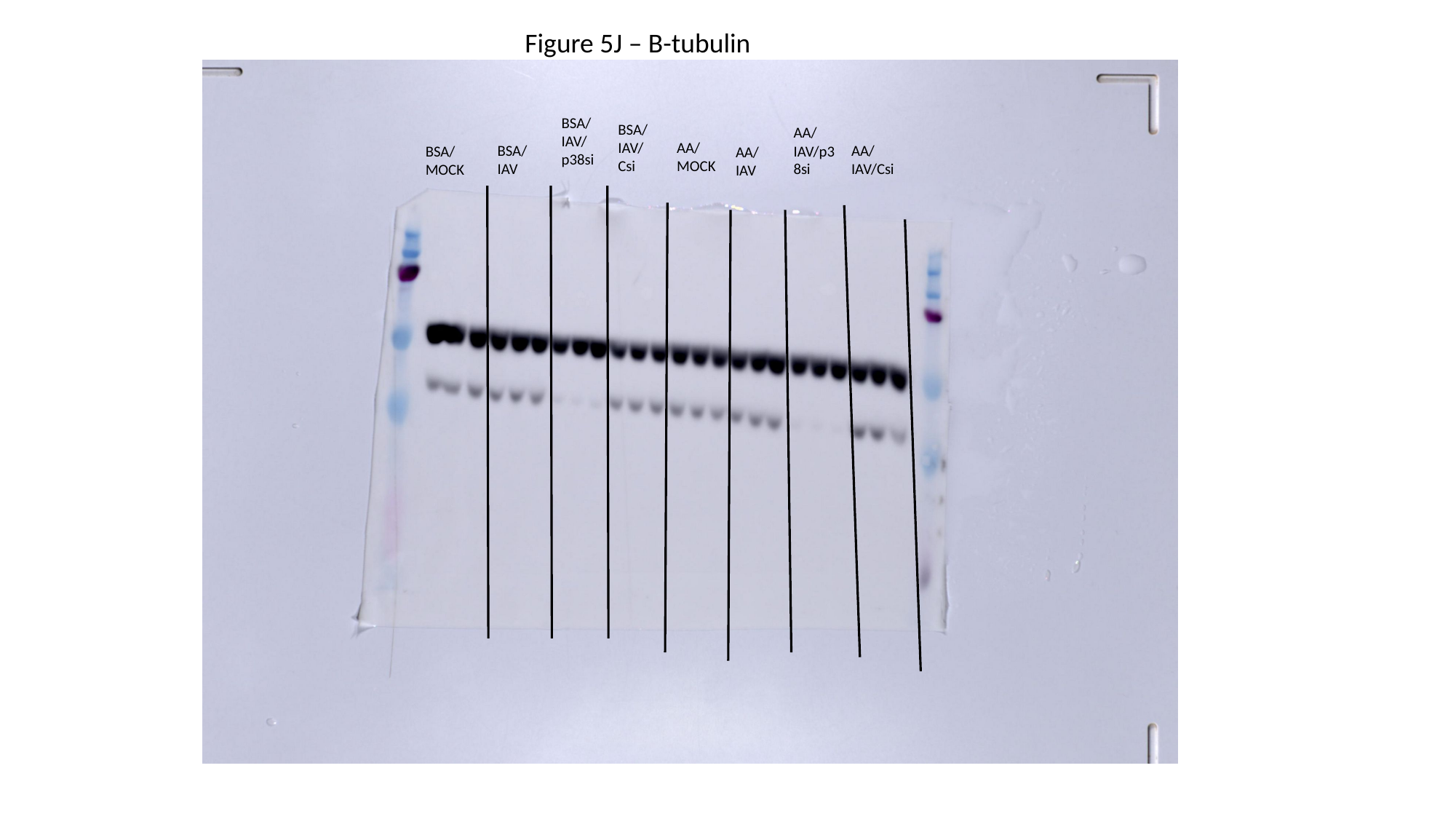

Figure 5J – B-tubulin
BSA/IAV/p38si
BSA/IAV/Csi
AA/ IAV/p38si
AA/ MOCK
BSA/IAV
AA/ IAV/Csi
BSA/ MOCK
AA/ IAV

## Slide 9
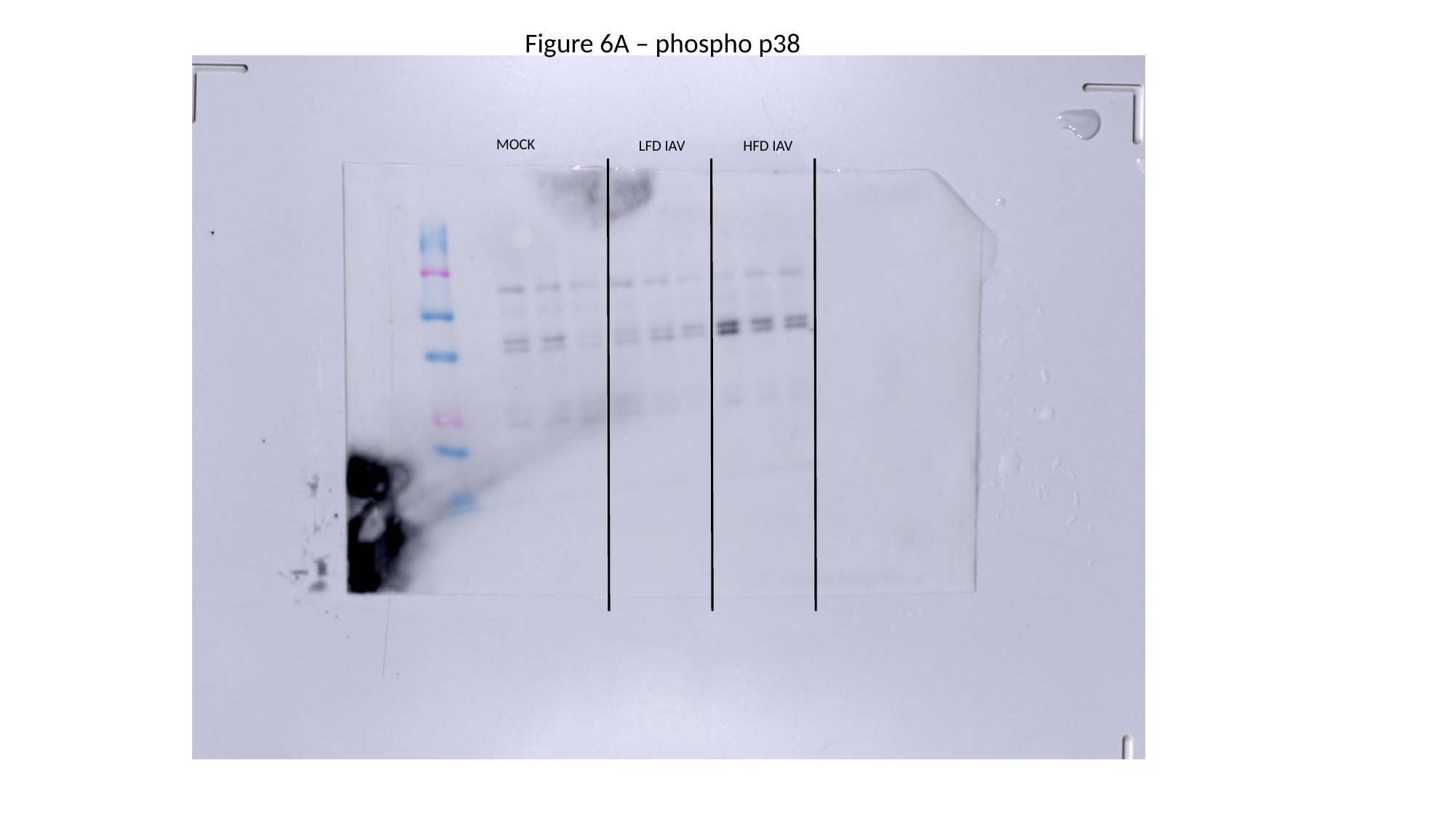

Figure 6A – phospho p38
MOCK
LFD IAV
HFD IAV

## Slide 10
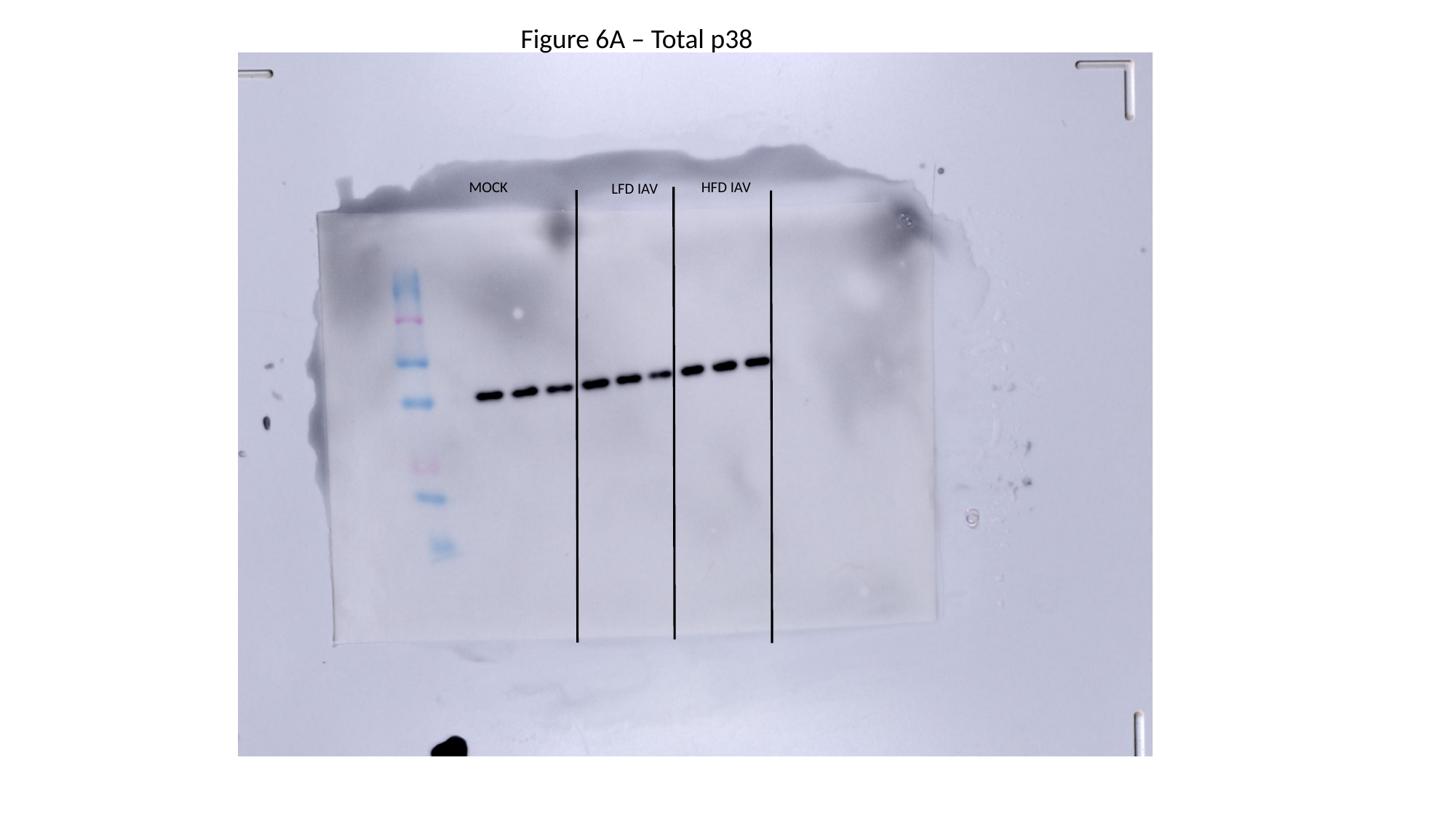

Figure 6A – Total p38
HFD IAV
MOCK
LFD IAV

## Slide 11
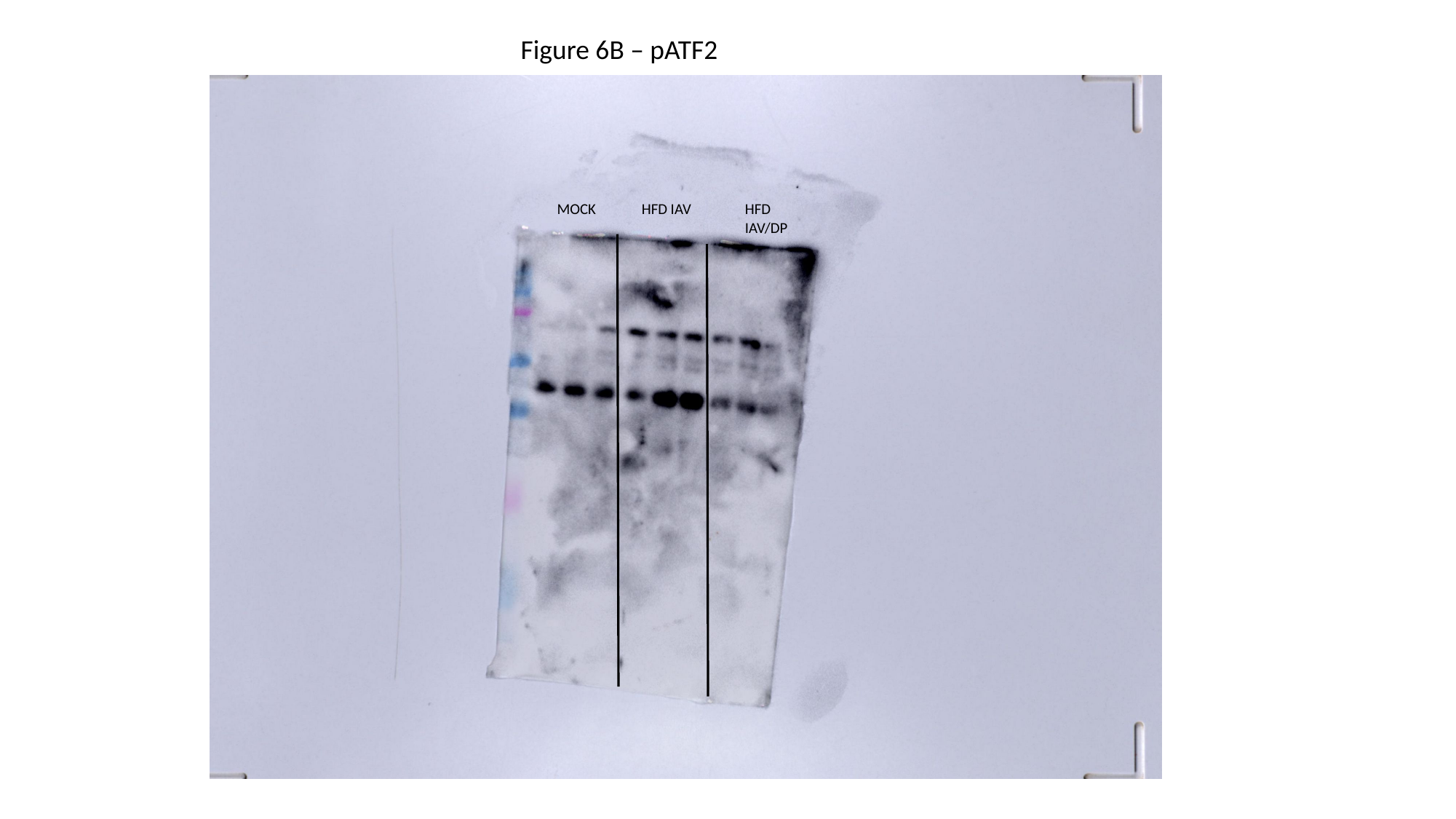

Figure 6B – pATF2
HFD IAV
HFD IAV/DP
MOCK

## Slide 12
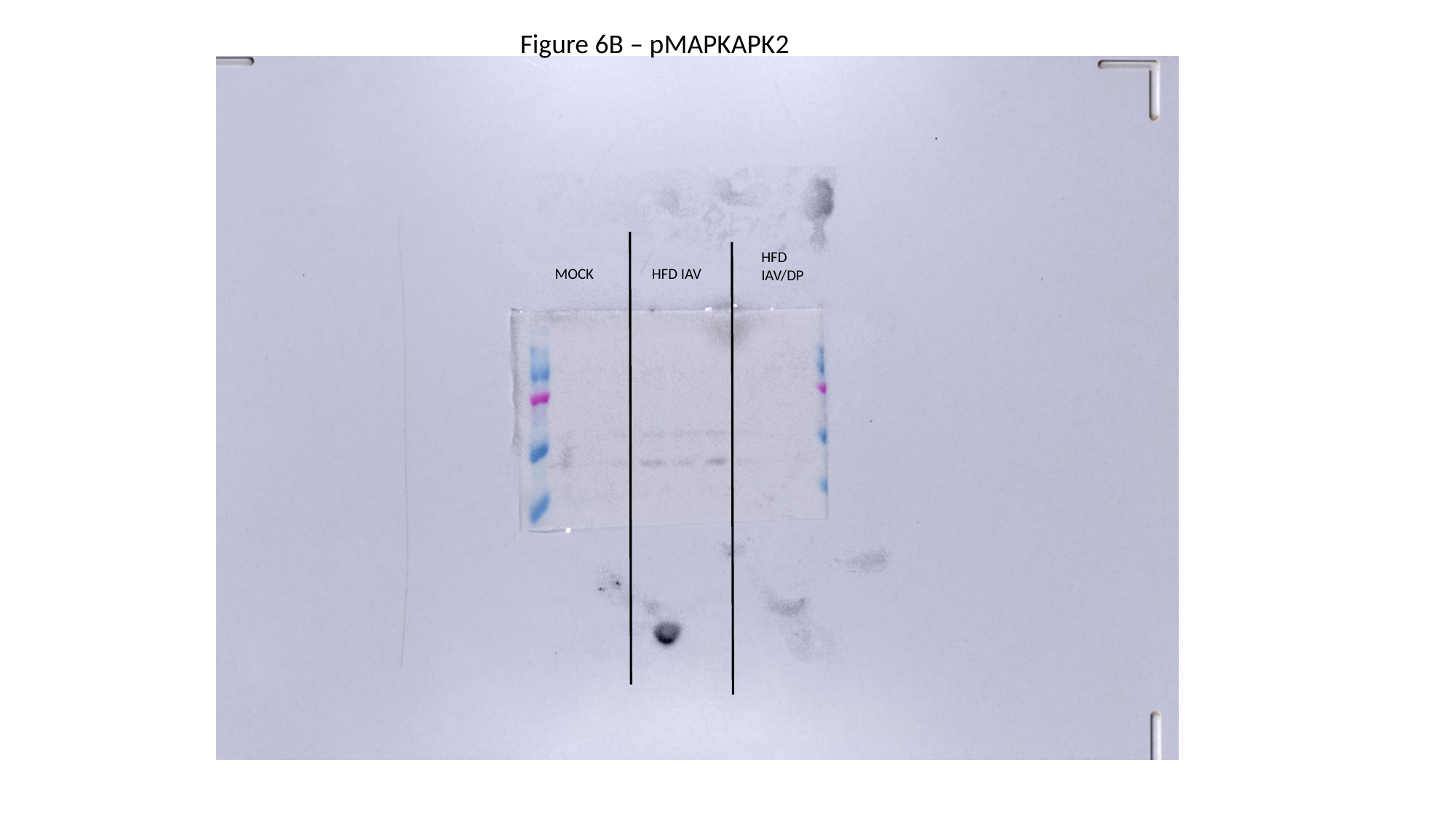

Figure 6B – pMAPKAPK2
HFD IAV/DP
HFD IAV
MOCK

## Slide 13
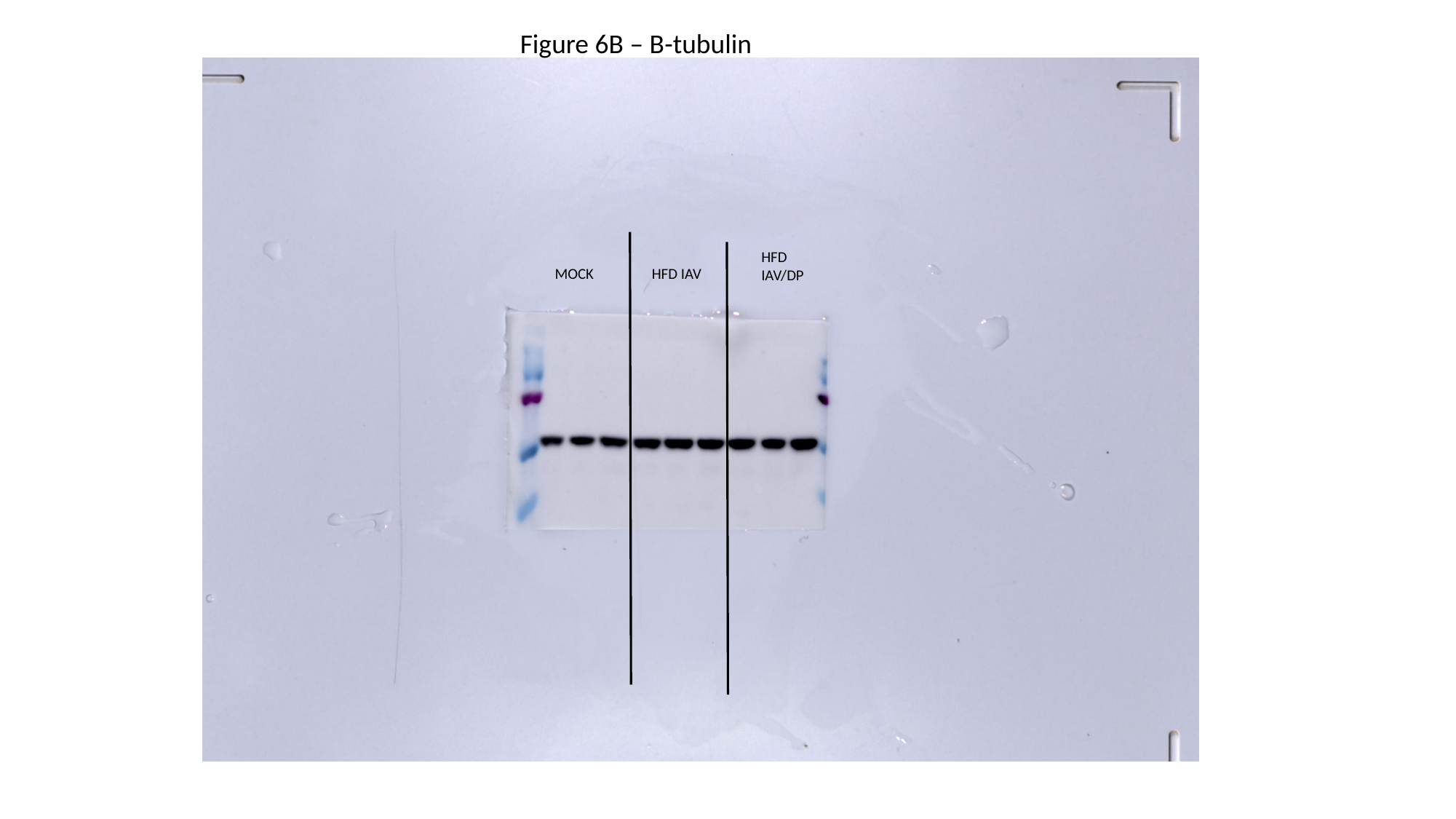

Figure 6B – B-tubulin
HFD IAV/DP
HFD IAV
MOCK
